# Variability in the Control of Cell Division Underlies Sepal Epidermal Patterning in *Arabidopsis thaliana*

**DOI:** 10.1371/journal.pbio.1000367

**Published:** 2010-05-11

**Authors:** Adrienne H. K. Roeder, Vijay Chickarmane, Alexandre Cunha, Boguslaw Obara, B. S. Manjunath, Elliot M. Meyerowitz

**Affiliations:** 1 Division of Biology, California Institute of Technology, Pasadena, California, United States of America; 2 Center for Integrative Study of Cell Regulation, California Institute Technology, Pasadena, California, United States of America; 3 Center for Advanced Computing Research, California Institute of Technology, Pasadena, California, United States of America; 4 Center for Bio-Image Informatics, Electrical and Computer Engineering Department, University of California, Santa Barbara, California, United States of America; John Innes Center, United Kingdom

## Abstract

How growth and proliferation are precisely controlled in organs during development and how the regulation of cell division contributes to the formation of complex cell type patterns are important questions in developmental biology. Such a pattern of diverse cell sizes is characteristic of the sepals, the outermost floral organs, of the plant *Arabidopsis thaliana*. To determine how the cell size pattern is formed in the sepal epidermis, we iterate between generating predictions from a computational model and testing these predictions through time-lapse imaging. We show that the cell size diversity is due to the variability in decisions of individual cells about when to divide and when to stop dividing and enter the specialized endoreduplication cell cycle. We further show that altering the activity of cell cycle inhibitors biases the timing and changes the cell size pattern as our model predicts. Models and observations together demonstrate that variability in the time of cell division is a major determinant in the formation of a characteristic pattern.

## Abstract 2

Live cell imaging and computational modeling explains how variability in the timing of cell division generates a characteristic pattern of cell sizes during development.

## Author Summary

How the regulation of cell division contributes to cell patterning in an organ is an important question in developmental biology. We chose to study cell size patterning in the *Arabidopsis* sepal, the green leaf-like floral organ, because it contains a wide range of cell sizes—from giant cells to small cells—and because sepals, as the outermost floral organ, are accessible for live cell imaging. In this study we image the early development of living sepals and follow each of the cell divisions to determine how cells of different sizes are created. We observe that the times when cells divide and when they stop dividing are highly variable. Using computational modeling, we then show that a model in which these decisions are made randomly with the probabilities we observed in vivo can recapitulate the production of the range of cell sizes seen in the living sepal. We also show that changing these probabilities within our model robustly predicts the novel cell patterns observed in mutant plants with altered cell division timing. We conclude that probabilistic decisions of individual cells—rather than deterministic, organ-wide mechanisms—can produce a characteristic and robust cell size pattern in development.

## Introduction

**Figure 1:**
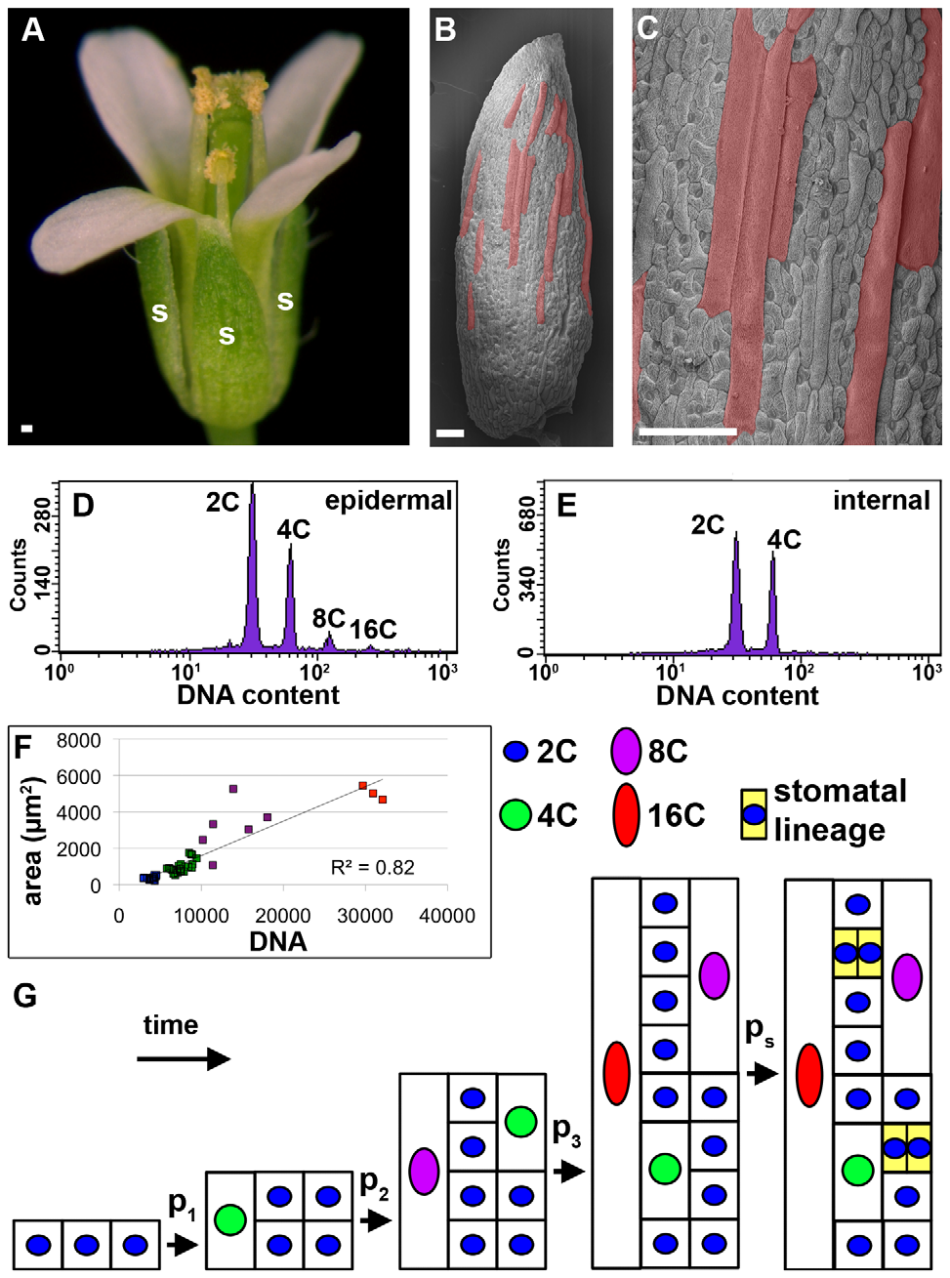
The cell size pattern in the *Arabidopsis* sepal epidermis. (A) Wild type *Arabidopsis* flower with sepals (s). (B, C) Scanning electron micrographs (SEMs) of a mature wild type sepal. Giant cells (false colored red) are interspersed between smaller cells. (D, E) Flow cytometric analysis of the DNA contents of nuclei in the mature wild type sepal epidermis (D, both front and back) and internal cell layers (E) used to derive the probability parameters for the model. Histograms show DNA content of each nucleus. (F) Graph of DNA content (integrated density of DAPI fluorescence) versus cell area (µm^2^) of mature sepal cells. The trend line of the data is displayed and *R* ^2^ = 0.82 (*n* = 47 pavement cells, normalized with fifty-nine guard cells which are known to be 2C). Ploidy of cells is indicated by color (red, 16C; magenta, 8C; green, 4C; and blue, 2C). (G) Conceptual model proposing that the probabilistic entry of cells into endoreduplication at different times generates the cell size pattern. Scale bars: 100 µm.

During development, complex patterns of specialized cell types emerge de novo. Pattern formation occurs in a changing environment where cells proliferate and differentiate, and we are interested in how regulation of cell division contributes to the patterning of an organ [1]. One system for investigating this problem is the development of the *Arabidopsis* sepal epidermis, which forms a characteristic cell size pattern ranging from giant cells stretching one fifth the length of the sepal to small cells stretching one hundredth the length of the sepal (Figure 1A–C; giant cells marked in red). The sepal is the outermost, green, leaf-like floral organ, which acts defensively to enclose and protect the developing reproductive structures. The sepals open at maturity when the flower blooms. Although the function of having a wide range of pavement cell sizes is unknown [2], it is possible that the diversity in cell sizes plays a role in defense against insect predators, helps the plant respond to water stress, or has a mechanical role (see Discussion). Within the flower, sepals are unique in containing such a pattern of diverse cell sizes and consequently giant cells have been used as a marker for sepal organ identity [3]–[6]. Outside the flower, a similar cell size pattern containing giant cells is found in the *Arabidopsis* leaf epidermis (Figure S2H) [7].

To understand the cellular basis of pattern formation, we need to investigate the development of the organ in real time with sufficient temporal and spatial resolution. When combined, three recent advances make this possible. First, by imaging living and developing tissues, it is possible to track individual cells and their divisions to determine the consequences of the division pattern on development [8]–[12]. Second, automated image processing can be used to extract quantitative data from images [13]–[15]. Third, computer modeling can be used to explore the consequences of temporally and spatially realistic biological hypotheses [13],[16]–[19] and can make predictions that can be tested with further imaging. In particular, many developmental models of multicellular plant tissues have been used to explore hypotheses about the role of transport of the plant hormone auxin in the shoot, root, and leaf primordia [20]. Models have also been used to predict the spacing of the hair cells in epidermis of the leaf (trichomes) and the root (root hairs); however, these models did not take into account the effect of cell division on the pattern [21],[22]. Several modeling strategies have been used to create multicellular structures including L-systems, dynamical grammars, cellular potts models, weak spring models, and finite element models [23],[24]. The combination of live imaging, image processing, and modeling are central to the computational morphodynamic approach to understanding plant growth [23]. In this study we use a computational morphodynamic approach to determine how the timing and position of cell division creates a specific pattern of cell sizes in *Arabidopsis*. We chose the sepal epidermis, instead of the leaf epidermis, as the system for addressing this question because sepals are easily accessible for live imaging and the cells are roughly rectangular, making the size distribution readily apparent for automated image processing.

The size of a cell is controlled by its growth rate and its frequency of division. Plant cells are confined by their cell walls [25], which cannot slide relative to a neighboring cell [26],[27]. Consequently within a layer such as the epidermis, it is unlikely that one cell can grow substantially faster than its neighbor. On the other hand, the frequency of division is regulated by the cell cycle. In the extreme case, cells enter the specialized endoreduplication cell cycle in which they replicate their DNA but fail to divide [28]–[30]. Consequently, endoreduplicating cells increase in both size and DNA content [7]. The principle that ploidy level, or the number of copies of the chromosomes, is generally correlated with cell size was first described as the karyoplasmic ratio about one hundred years ago by Boveri, Hertwig, and their colleagues [31],[32]. Since then, the constancy of the ratio between DNA content and cytoplasmic volume had been demonstrated in various cell types including epidermal cells [7] and hair cells (trichomes) of *Arabidopsis* [33],[34] as well as nearly every organism from bacteria [35] to mammals [31],[36]. The disruption of many *Arabidopsis* cell cycle regulators has been shown to affect both endoreduplication and cell size (reviewed in [37]); however, these studies have examined the average responses of a whole population of cells and have not been able to resolve the timing of the responses of individual cells or how these individuals together generate a pattern [38].

Here we ask how the temporal regulation of cell division, endoreduplication, and growth combine to create the pattern of giant cells and small cells in the sepal epidermis. We use live imaging to determine the timing and position of each cell division in the outer (abaxial) sepal epidermis and track the lineages of these cells throughout early sepal development. This information is used to create a computational model that captures the essential aspects of the dynamics of epidermal pattern formation and reproduces them *in silico*. The model is then tested against the measured in vivo cell size distribution. We show that the distribution of cell sizes is formed through variability: probabilistic decisions to enter endoreduplication, noise in the duration of the cell cycle, and variation in the daughter cell sizes. Finally, we show that the model is predictive in that changing model parameters generates the phenotypes of mutants.

## Results

### Quantitative Characterization of the Sepal Cell Size Pattern

The largest group of cells in the outer (abaxial) sepal epidermis of *Arabidopsis* appears to be a distinct class in that they bulge from the plane of the epidermis (Figure 1A–C). We designate these as giant cells (Figure 1B–C). Giant cells average 11,000 µm^2^ (±5,400 µm^2^ s.d.) in area and 360 µm (±150 µm s.d.) in length (*n* = 62). The longest giant cells can reach 800 µm. The giant cells are interspersed between smaller cells, which are level with the plane of the epidermis. Both giant cells and small cells have a range of sizes and constitute the pavement cells, which are the primary epidermal cell type that serve to form a protective barrier for the organ. In addition the epidermis contains hair cells (trichomes, which are not present on all sepals) and guard cells, which constitute 29% (±3% s.d., *n* = 12 sepals) of the cells in the outer sepal epidermis. Guard cells surround the stomatal pores and regulate gas exchange [39].

Large cells in plants are typically highly endoreduplicated; however, Galbraith et al. had previously found that floral buds do not contain endoreduplicated cells [40]. To address this discrepancy, we used flow cytometry to measure nuclear DNA content in mature sepals. Only non-endoreduplicated nuclei were detected in the internal cells of the sepal (2C and 4C), indicating that endoreduplication occurs in the epidermis where 1.0%±0.01% of the cells are 16C and 5.5%±1.4% of the cells are 8C (Figure 1D–E; ±95% confidence interval, *n* = 5 replicates totaling 31,744 nuclei). This result parallels the leaf epidermis where the giant cells are also 16C [7] indicating that in both leaves and sepals, pavement cells can undergo as many as three endocycles. We next verified that cell size largely correlates with DNA content in sepals as had been observed in leaves [7]. DNA content correlates directly with cell area with an R-squared correlation value of 0.82, indicating that about 80% of the variation in cell size can be explained by differences in endoreduplication (Figure 1F). Giant cells are primarily 16C, although occasionally 8C giant cells are found. Therefore endoreduplication is a primary determinant of cell size and we looked for mechanisms through which endoreduplication can control the cell size pattern.

### Patterning as a Result of the Division of Space

We first drew a conceptual model to predict the cell division pattern that gives rise to the pattern of giant cells and small cells within a small region of the sepal (Figure 1G). Growth and cell division in the epidermis are constrained to maintain a single clonal cell layer [41]. Previously, Traas et al. had hypothesized that in such a constrained tissue, cell size is controlled through the timing of endoreduplication [2]; however, this hypothesis has not been tested. If all of the cells grow at the same rate, then the earlier a cell stops dividing and enters endoreduplication, the larger it becomes while the other cells continue to divide, thus retaining their small size. We hypothesize that after a mitotic division period each cell has three cell cycles, which can be either mitotic or endocycles (Figure 1G). We term these three cell cycles the patterning cell cycles since they will generate the cell size distribution. We further hypothesize that the decision of each cell to endoreduplicate is random and is governed by a probability that may change in time (Figure 1G). At the first cell cycle, each cell makes the random decision with probability p_1_ to endoreduplicate to 4C and double its area, or with probability 1-p_1_ to divide and remain 2C. Once a cell has decided to endoreduplicate, it continues to endocycle and cannot resume mitotic divisions [42], although exceptions in specialized circumstances have been observed [43]. Therefore, in the next cell cycle, all of the 4C cells endoreduplicate to 8C and grow to 4 times their original area. Simultaneously, the 2C cells decide to endoreduplicate to 4C with probability p_2_ and the remaining cells divide. In the final patterning cell cycle, those cells that decided to endoreduplicate earliest become the 16C giant cells. The remaining 2C cells decide to endoreduplicate with probability p_3_ or divide and become the smallest cells. The specialized division patterns of stomatal development follow at the end of this process and 2C cells continue to divide with probability p_s_ (Figure 1G). In this model, the timing of entry into endoreduplication generates the spatial pattern of cell size within a small region of the sepal.

### Timing of Endoreduplication Correlates with Cell Size

**Figure 2:**
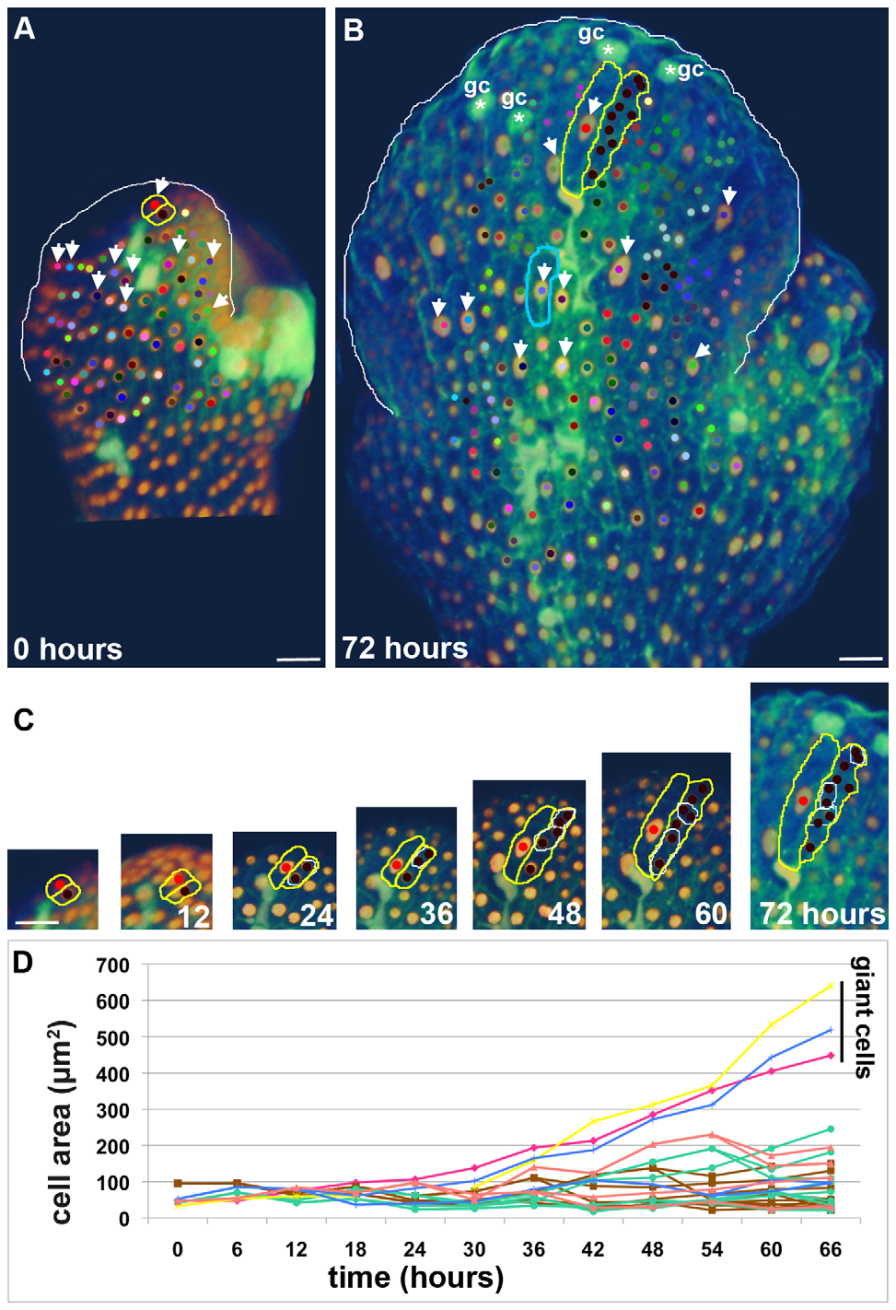
Cell size correlates with timing of endoreduplication. (A, B) Live imaging of the development of a wild type sepal primordium imaged every 6 h for 72 h corresponding to Video S1. Images show epidermal nuclei (*pATML1::H2B-mYFP*) in gold and cell walls (propidium iodide [PI]) in green. A cell and all of its progeny receive the same colored dot. The sepal is outlined in white, giant cells that fail to divide throughout the sequence are indicated with white arrows, and differentiated guard cells (gc) are indicated with a white asterisk. An example of neighboring giant cell and small cell clones are outlined in yellow (shown in detail in C). Note that these clones grow to the same extent. However, comparing the giant cell outlined in yellow to the giant cell outlined in blue shows that growth throughout the sepal is not equivalent. (C) Tracking the development of the neighboring red giant cell and the brown small cell lineage (outlined in yellow in A–B). Images are at the same magnification. The daughter nuclei resulting from a division are circled in white. Although the red and brown cells are equivalent in size and appearance in the young sepal primordium, the red cell never divides throughout the 72-h sequence and becomes a giant cell. By 48 h the red cell nucleus has started to enlarge, indicating that the cell is endoreduplicating. In contrast, the brown cell progeny have undergone two, three, or four divisions. (D) Graph showing that the area of cells depends on the division pattern. The areas of six progenitor cells (outlined with plasma membrane marker *pATML1::mCitrine-RCI2A*) from Video S3 (labeled with equivalent colors) were tracked over time showing that cells generally increase in size except when they divide. Note the daughter cell sizes are often not exactly equal and that the cell cycle times are nonuniform. For more details on small cell lineages, see Figure S1. Scale bars: 20 µm. See also Videos S1, S2, S3.

To test our hypothesis that timing of endoreduplication determines the final cell size, we tracked cell divisions and endoreduplication in living sepal primordia (Figure 2). Live imaging confirms that giant cells stop dividing and begin endoreduplicating in the young sepal primordium while smaller cells continue to divide as predicted (*n* = 109 lineages from 3 sepals; Figure 2; Videos S1–S3). In the initial time frame just after the sepal primordia are formed, all of the nuclei are small, falling into approximately two size categories, indicating that all cells are still 2C or 4C depending on their stage in the cell cycle (Figure 2A and C). In plant cells, nuclear size is highly linearly correlated with DNA content [42],[44], and the expression of the H2B-YFP fusion protein has been shown to have no effect on cell cycle dynamics [45]. From this early time point the giant cells never divide and their nuclear size increases, indicating that they are endoreduplicating (Figure 2B arrows). Generally we find that endoreduplicating cells do not divide, consistent with our hypothesis, but on rare occasions an endoreduplicating cell has been observed to divide (Video S3). During this same 3-d period, the smaller cells undergo primarily one to four rounds of division although a few cells undergoing five rounds are present (Figure 2C). This is consistent with the hypothesis that small cells have only three patterning cell cycles plus a couple of stomatal divisions. The differentiation of stomata is observed near the top of the sepal at the end of the 3-d period (Figure 2B).

To confirm that the cell size corresponds with the division pattern, we tracked the cell size in six lineages (Figures 2D and S1; Video S2). Through the first 24 h the sizes of all the cells are relatively uniform. After 24 h, the size of the giant cells steadily increases as they endoreduplicate. In contrast, each time a cell divides the volume is split, resulting in two smaller cells in the small cell lineages.

### Computational Modeling Reproduces the Cell Size Pattern of the Whole Sepal

**Figure 3:**
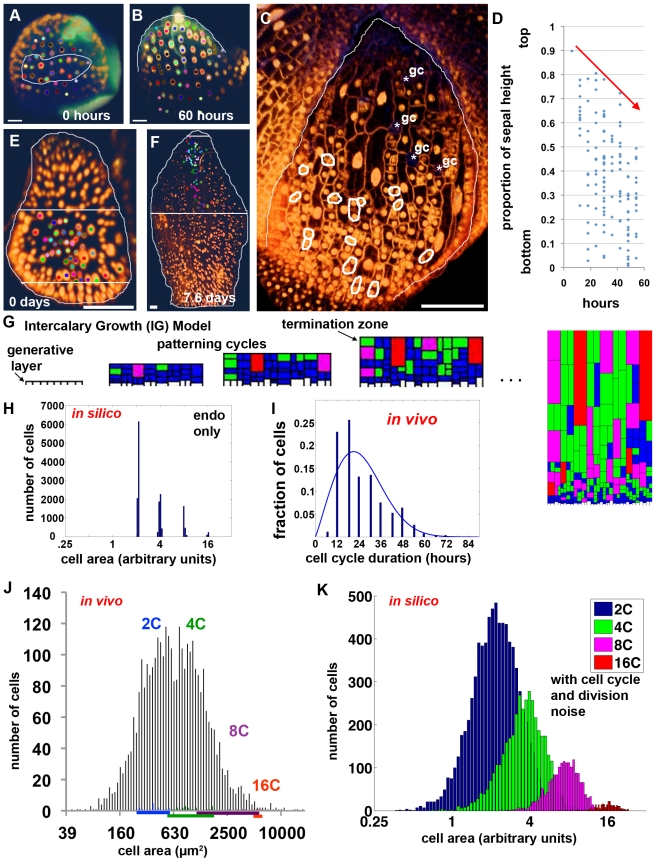
The Intercalary Growth Model reproduces the cell size distribution. (A, B) Live imaging of the initiation of a wild type sepal primordium showing that based on lineage analysis the outer sepal epidermis is derived from approximately two rows of 8 cells (outlined in white) (Video S5). Epidermal nuclei (*pATML1::H2B-mYFP*) are in gold and cell walls (PI) are green. Each cell and all of its progeny are labeled with the same colored dot. A bright dot sitting on the top of the sepal in (B) is a pollen grain. Scale bars: 10 µm. (C) Single frame from live imaging of a lateral sepal (outlined in white) (Video S6). Plasma membranes are also marked in gold (*pATML1::mCitrine-RCI2A*). Note the top of the sepal contains differentiated guard cells (asterisk gc) and the cells are no longer dividing, whereas cells in the bottom of the sepal are actively dividing (daughters of divisions occurring within the last 6 h are circled in white). Scale bar: 50 µm. (D) Scatter plot showing the vertical position of each division (Video S6) event as a percentage of the sepal length (1 = top and 0 = bottom). Red arrow indicates the progressive basipetal termination of divisions. (E–F) Live imaging of an older sepal primordium imaged every 12 h (E: day 0) through maturity at stage 12 (F: 7.6 d). Cells from the middle of the sepal primordium and their progeny have been tracked throughout the 7-d sequence and have been used to delineate regions of the primordium and corresponding regions of the mature sepal (outlined in white). These regions are arbitrary and depend only on the cells that were chosen for tracking. Note that the top half of the primordium makes only the tip of the mature sepal, whereas the middle of the primordium makes the top half of the sepal, and the bottom few cell layers make the whole bottom half of the mature sepal, indicating that these bottom cells have continued to proliferate. Scale bars: 50 µm. (G) Intercalary Growth (IG) Model. The computational sepal develops from an oversimplified generative layer of 8 cells. In the model, the generative layer cells proliferate throughout development. The upper progeny of the generative layer enter the patterning divisions and terminate after undergoing three cell cycles whether mitotic or endocycles. The final image to the right is at a reduced magnification. Cell color corresponds to ploidy: white, 2C generative layer; blue, 2C; green, 4C; magenta, 8C; and red, 16C. (H) Histogram showing that cells are produced in four exact sizes by the IG model when only endoreduplication is allowed to vary. Cell cycle lengths are constant and divisions are exactly symmetric (compare with panel K). The area axis is scaled at log base 2. (I) Histogram of cell cycle times (in 6-h increments) measured from the wild type live imaging data (Videos S1, S2, S3). (J) Histogram of cell areas in the mature sepal epidermis determined by semi-automated image processing (grey; see Text S1 for details). The area axis is scaled at log base 2. The ploidy of cells is calibrated with the 47 pavement cells for which both DNA content and area are known from Figure 1F (the extent of the region is underlined). Note that cells areas fall in a broad distribution although peaks for 2C and 4C are visible. (K) Histogram of cell areas produced by the IG Model, including variability in the cell cycle time and noise in the symmetry of division, showing that each ploidy level has a distribution of cell sizes. The overall size distribution is not significantly different from the in vivo distribution (Figure 3J) (see Text S1 for further analysis). See also Videos S4–S6.

We developed a computational model to reproduce the cell size pattern of the approximately 1,600 cells (Figure S2J) in the wild type sepal epidermis (Figure 3G, Video S4). Although the conceptual model (described above, Figure 1G) reproduces a local area of the sepal, without a very large starting population of cells, it fails to produce the whole organ. To determine how the complete pattern is formed, we used additional live imaging experiments as the basis for the creation of the Intercalary Growth Model (IGM) (see Text S1). First, we imaged the emergence of the sepal primordium from the floral meristem to determine the number and arrangement of cells, which was used as the basis for the cellular template (initial geometrical structure of cells) for the model. The sepal initiates from a region on the lateral side of the floral meristem that is approximately 8 cells wide (Figure 3A,B; Video S5; *n* = 2 sepal primordia). This result is consistent with sectoring data that suggested the sepal emerges from a file of 8 cells on the floral meristem [46]. Therefore, we initiate the model as a file of 8 cells (Figure 3G).

The differentiation of stomata and the termination of cell divisions progress in a wave from the top to the bottom of the sepal as have been previously observed in the leaf [47],[48]. The top cells stop diving while cells in the remainder of the sepal continue to proliferate (Figure 3C–D; Video S6). Furthermore, the cells in the whole top half of the young sepal primordium generate only the very tip of the mature sepal (Figure 3E,F) suggesting that they have undergone few further divisions. Cells originating from the lower middle of a young sepal primordium form the top half of the sepal as they undergo the patterning divisions to create giant cells and small cells. In contrast the bottom cells in the sepal primordium proliferate to give rise to the whole bottom half of the mature sepal, indicating that they continue proliferative mitotic divisions and only enter the patterning division stage later.

We incorporate this wave of progressive maturation into the IGM model by allowing the basal layer of cells, which we call the generative layer, to proliferate giving rise to apical daughters or additional generative layer cells throughout sepal development (Figure 3G), similar to the concept of the intercalary meristem of grass leaves [49]. This generative layer starts as a file of 8 cells as determined by imaging the initiation of the sepal. Although the generative layer is an oversimplification, a population of cells within the base of the sepal generally maintains their ability to proliferate. In the model, the upper daughter cells of the generative layer enter the patterning cell divisions as described in the conceptual model, giving these cells three cell cycles in which to divide or endoreduplicate plus entry into stomatal development (Figures 1G, 3G; Video S4; stomatal development is not modeled). To find the probabilities with which cells enter endoreduplication at each cell cycle (p_1_, p_2_, and p_3_) we fit a population model similar to [50] (see Text S1 for details) to the final distribution of endoreduplicated cells as determined by flow cytometry (Figure 1D). The probability of endoreduplicating increases as cell cycles progress from a low value of p_1_ to a high value of p_3_ (see Text S1). For the cells that divide, only horizontal or vertical division planes are allowed. The plane is chosen that produces daughter cells with the length to width ratio closest to 2∶1. As a result cells divide in both orientations in the model (Video S4), similar to the living sepal where both planes of division are commonly observed (Videos S1–S3). At the end of these three patterning cell cycles, the cells stop dividing, stop growing, and their size is measured. Although these cells have stopped growing, in the visualization of the model, they continue to expand due to constraints of the geometrical model (see Text S1). Thus the wave of termination in cell division observed arises naturally from the model as the upper cells terminate after their cell cycles while the subsequent progeny of the generative layer simultaneously start their patterning cell cycles (Figure 3G). Sepal development progresses until about 1,600 cells are produced, similar to wild type sepals (Figure S2J). We conclude that repeating the patterning process with each new set of cells arising from the generative layer appears similar to a sepal in that the wave of proliferation terminates from the top. Furthermore, the model produces a sufficient number of cells and we next determined whether the cell size pattern produced matches the in vivo pattern.

### Unequal Divisions and Asynchronous Cell Cycles Generate Variability in Cell Size

We tested whether the model can predict the cell size distribution in the sepal epidermis. If only the probability of endoreduplication is included, the IGM model produces four discrete cell sizes, one for each cell ploidy in the sepal epidermis (Figure 3H). These results correspond with our conclusion that the timing of endoreduplication and hence the number of endocycles completed is the major determinant of cell size.

To test whether the sepal cells fall into four strict cell size categories as predicted by the model, we measured the in vivo cell areas through semi-automated image processing. This dataset was not used in the generation of the model and is therefore an independent test of the model. In this dataset, the areas of pavement cells range from 45 µm^2^ to 17,414 µm^2^ with no clear distinct size classes although presumptive 2C and 4C peaks are visible (*n* = 3,295 cells from 12 sepals) (Figure 3J). The higher level of cell size variability observed in real sepals than produced by the model when only endoreduplication is allowed to vary (Figure 3H) suggested that although endoreduplication is the primary determinant of cell size, other factors also contribute to the distribution of cell sizes around the mean established for each ploidy level.

To identify the factors that contribute to variability in cell size, we reexamined the wild type live imaging results (Figure 2). First, contrary to the original assumptions of the hypothesis, live imaging shows that the lengths of the cell cycles are not equal and divisions are not synchronous (Figure 2C–D; Figure S1). For example, the two brown daughters at 24 h have divergent cell cycle times (Figure 2C). The upper daughter divides twice by 48 h, while the lower daughter divides once by 60 h. Throughout this longer cell cycle, the bottom daughter grows and reaches a larger size before division than the upper neighbors with faster cell cycles, indicating that cell cycle time is one of the primary factors that add variability to the cell size around the mean established by the ploidy of the cell. We measured the distribution in cell cycle times from the live imaging data and found that it was a broad distribution (Figure 3I). We sampled cell cycle times in the IGM from this distribution to reproduce the asynchrony observed in vivo (Video S4; see also Text S1). As a result, for each ploidy the *in silico* cells form a distribution around the mean cell size (Figure 3K).

Second, contrary to the original assumptions of the hypothesis, normal pavement cell division does not generally produce daughter cells with precisely equal areas. The standard deviation in daughter cell sizes is 8.5%. In addition, the divisions in the stomatal lineage are highly asymmetric as previously described (Figure S1) [39]. Therefore we select the cell division plane in the IGM to create daughter cells with a 10% standard deviation in areas (Figure 3G; Video S4).

With asynchronous cell cycles and slightly unequal divisions, the IGM produces a wide distribution of cell sizes similar to that of the in vivo sepal (Figure 3K). If additional divisions to represent the stomatal lineage are included, the cell size distribution produced by the model is not significantly different from in vivo cell size distribution, indicating that the simple rules of the model are sufficient to predict the in vivo cell size pattern (see Text S1 for statistics).

### Using the Model to Predict the Development of Plants with Altered Cell Size Patterns

**Figure 4:**
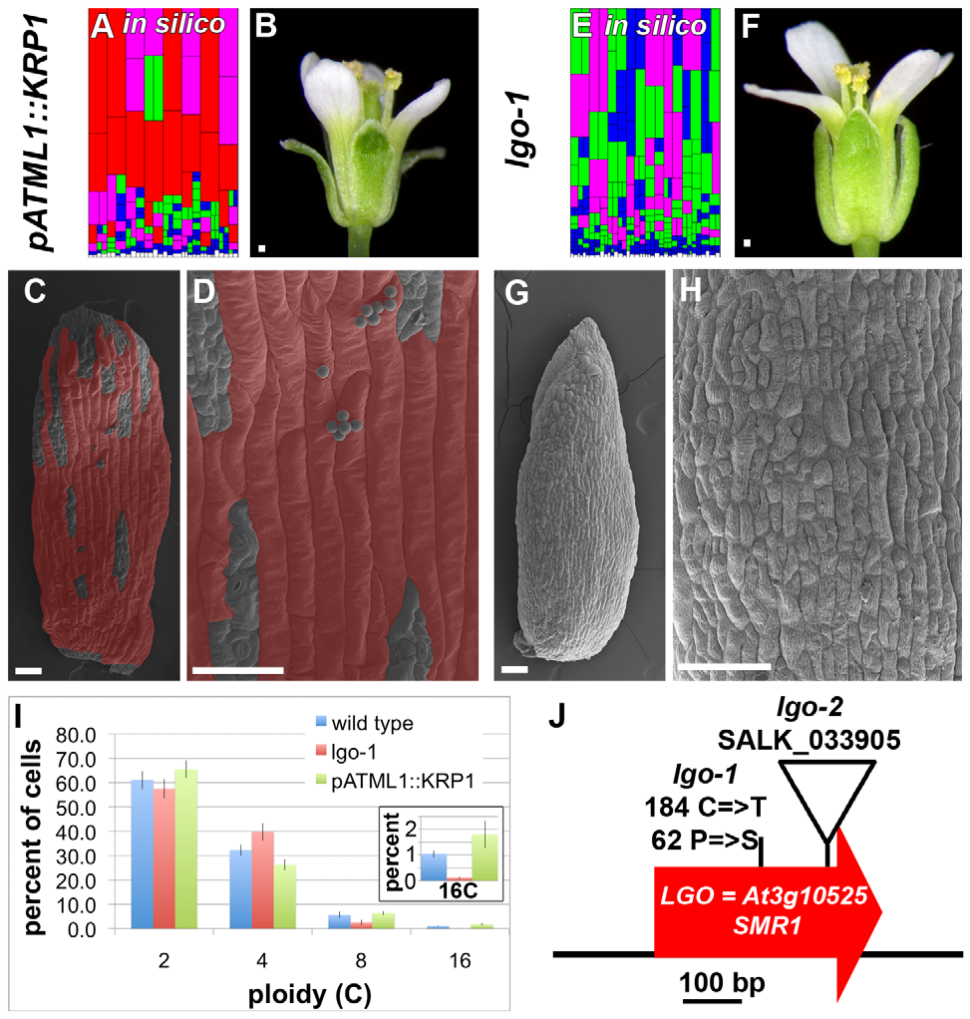
The model predicts the phenotypes of plants with altered cell size distributions due to gain or loss of cell cycle inhibitor function. (A) Increasing the probability of entering endoreduplication (p_1_ = 0.5) in the first cell cycle of the IG model creates sepals with additional giant cells similar to the phenotype of *pATML1::KRP1* sepals. Compare to Figure 3G. Cell color corresponds to ploidy: white, 2C generative layer; blue, 2C; green, 4C; magenta, 8C; and red, 16C. (B) *pATML1::KRP1* flower. Note the abnormal outward curvature of the sepals. (C, D) SEMs of a *pATML1::KRP1* sepal showing giant cells interrupted by islands of small cells. Giant cells are false colored red. (E) Setting the probability of entering endoreduplication in the first cell cycle to zero (p_1_ = 0) in the IG model creates sepals without giant cells similar to *lgo* sepals. Compare with Figure 3G. (F) *lgo-1* mutant flower. (G, H) SEMs of a *lgo-1* sepal showing the absence of giant cells. (I) Flow cytometry analysis of the epidermal DNA content in wild type, *lgo-1*, and *pATML1::KRP1* sepals. The inset shows an enlargement of 16C graph. Graph shows mean percentages and error bars represent the 95% confidence interval. (J) *LGO* encodes the cell cycle inhibitor SIAMESE RELATED 1 (SMR1) (AT3g10525). The *lgo-1* allele contains a mutation of C to T at base 184, which causes substitution of serine (S) for proline (P) at amino acid 62. The *lgo-2* allele contains a T-DNA insertion. Scale bars: 100 µm. See also Videos S7, S8 and Figure S2.

To further test the model, we used it to make two predictions about how changing the parameters affects the cell size pattern. First, when we increase the probability of entering endoreduplication at the first cell cycle, p_1_, more cells enter endoreduplication early and consequently the resulting simulated sepals have more giant 16C cells at the expense of small cells (Figure 4A and Video S7). Second, at the other extreme, if we set p_1_ equal to 0, all of the cells divide in the first cell cycle and the model produces sepals with no giant 16C cells but a distribution of the smaller cell sizes (Figure 4E and Video S8). Based on these results we predict that a dramatic alteration in the cell size pattern should reflect a change in the probability of endoreduplication at a given time.

### Early Endoreduplication Promotes Giant Cell Development

To test the first prediction biologically, we identified plants with an altered cell size pattern in the sepal epidermis. When the cell cycle inhibitor *KIP RELATED PROTEIN1* (*KRP1*) is moderately overexpressed in the epidermis (*pATML1::KRP1*), numerous giant cells form in the sepals (Figure 4B–D compare to Figure 1A–C) [51]. While strong expression of cell cycle inhibitors in the KRP family has been shown to block the cell cycle entirely, moderate levels of KRP overexpression result in increased endoreduplication [52]. *pATML1::KRP1* sepals contain 1.8% (±0.5% s.d.) 16C cells, nearly double the percentage in wild type sepals (1.0%±0.1% s.d.) (Figure 4I). Although the *pATML1::KRP1* sepals appear to be covered with giant cells, the actual number of giant cells is roughly doubled, which matches the increase in ploidy observed through flow cytometry. The large size of the giant cells means that a small increase in cell number causes giant cells to cover much of the area of the sepal.

**Figure 5:**
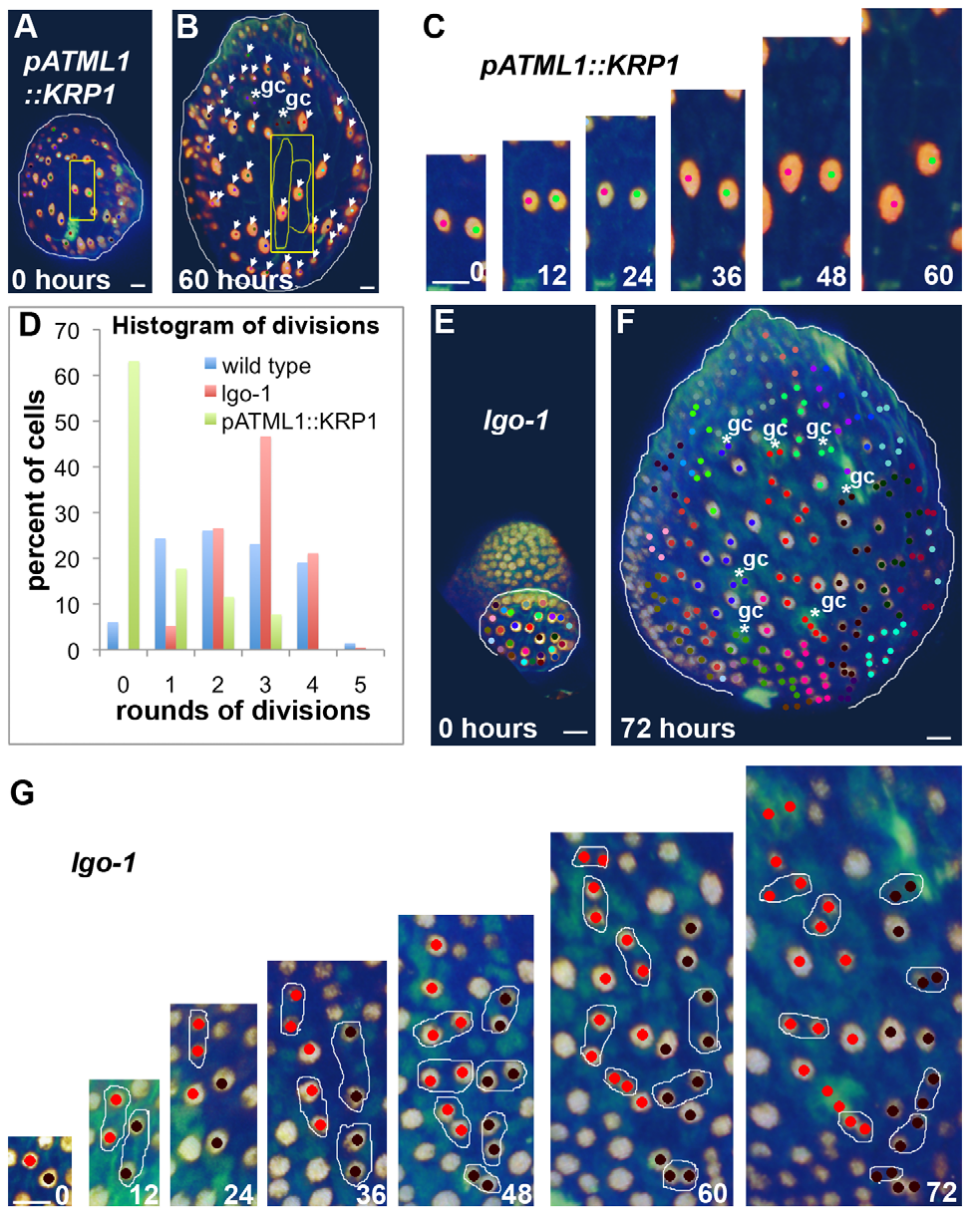
Cell cycle inhibitors promote early entry into endoreduplication. Epidermal nuclei (*pATML1::H2B-mYFP*) are in gold and cell walls (PI) are green. Each cell and all of its progeny are labeled with the same colored dot. Cells that do not divide throughout the image sequence are marked with white arrows and guard cell pairs (gc) are noted with white asterisk. Sepals are outlined in white. Compare with Figure 2. (A–C) Live imaging of a *pATML1::KRP1* sepal for 60 h (Video S9). (C) Two endoreduplicating cells grow throughout the time series. (D) Graph comparing the number of rounds divisions undergone by wild type, *pATML1::KRP1*, and *lgo-1* cells during the imaging sequences. Cells that have undergone four or five rounds of division are generally in the stomatal development pathway. (E–G) Live imaging of *lgo-1* for 72 h (Video S12). (G) All the cells continue dividing through 48-h time point, whereas in wild type the giant cells have already stopped dividing at the time point equivalent to 12 h. Scale bars: 10 µm. See also Videos S9–S14.

We then asked whether the time at which cells start to endoreduplicate was altered in real *pATML1::KRP1* sepals. Live imaging of *pATML1::KRP1* sepals revealed that a larger fraction of the cells stopped dividing and started endoreduplicating at early stages of sepal development corresponding to an increase in the probability of entering endoreduplication (Figure 5A–D; Videos S9–S11). The nuclei in *pATML1::KRP1* sepal primordia appear enlarged earlier than wild type giant cell nuclei do, suggesting entry into endoreduplication occurs earlier in *pATML1::KRP1* than wild type (Figure 5A,C). We conclude that promoting early endoreduplication through overexpression of the cell cycle inhibitor KRP1 produces increased numbers of giant cells in the pattern as predicted by the computational model.

### Delayed Endoreduplication Promotes Small Cell Formation

To isolate plants at the other extreme of the cell size distribution corresponding to the second prediction of the model, we conducted a mutagenesis screen for plants lacking giant cells in the sepals (see Supplemental Procedures Text S1). One of the mutants isolated was named *loss of giant cells from organs* (*lgo*) because the giant cells in both leaves and sepals were absent (Figure 4F–H compare to Figure 1A–C; Figure S2H–I). 16C cells are absent in the *lgo* sepal epidermis and the proportion of 8C cells is reduced (Figure 4I). We conclude that the *LGO* gene is necessary for giant cell formation in the plant.

As a direct test of the model we asked whether all *lgo* cells continue to divide throughout early development and enter endoreduplication later than wild type as predicted. The model passes the test since live imaging shows that all of the cells in the *lgo* sepal divide at least once after wild type giant cells have stopped dividing, indicating that entry into endoreduplication is delayed (Figure 5D–G; Videos S12–S14). The division patterns of all of the cell lineages of *lgo* appear similar to the wild type small cell lineages (Figure 5G compared with Figure 2C).

Through positional cloning we found that the *LGO* gene encodes a member of a plant specific cell cycle inhibitor family (SIAMESE RELATED 1 At3g10525) (Figure 4J; Figure S2A–D; see Text S1 for details) [53]–[55]. SIAMESE, the founding member of this cell cycle inhibitor gene family, promotes endoreduplication in the hair cells (trichomes), which are another highly endoreduplicated epidermal cell type (32C–64C) [53],[55]. In *siamese* mutants, the hair cells divide when they should endoreduplicate, creating multicellular hair cells [55]. This phenotype parallels the loss of giant cells in *lgo* due to extra divisions instead of endoreduplication. Consistent with the tradition of renaming gene family members when the function is determined [56],[57], we hereby rename the *SIAMESE RELATED 1* gene as *LGO*. *LGO* is broadly expressed throughout the plant [53]. Overexpression of *LGO* produces a phenotype similar to *pATML1::KRP1* with additional giant cells (Figure S3). Taking the results on the loss of *lgo* function and the gain of *KRP1* function together, we conclude that cell cycle inhibitors are important for setting the timing of entry into endoreduplication and consequently the cell size pattern.

It is unclear whether LGO promotes the early entry of pavement cells into endoreduplication only at the first cell cycle or promotes endoreduplication at all cell cycles. A higher proportion of 4C cells are found in *lgo* sepals (Figure 4I); however, the class of 4C cells includes both cells that have endoreduplicated and mitotic cells in the G2 phase after DNA replication of the cell cycle. Previous work suggests that the extra 4C cells in *lgo-1* mutants are likely to be mitotic cells that are in G2. Cell cycle inhibitors including LGO are postulated to bind to and inhibit CYCLIN D CDKA;1 complexes that regulate the transition from G1 to S phase [53],[54],[58],[59]. In the absence of *lgo*, the lack of this inhibition might be expected to increase the speed of entry into S phase of the cell cycle and consequently a greater fraction of the population of cells would be in G2, adding to the 4C population. A similar result was seen due to the overexpression of *CYCLIN D3;1*, which increased the number of 4C cells, and these cells were determined to be cells in G2 [60],[61]. In any case, the functional effect of the *lgo-1* mutation is to delay endoreduplication, which causes an absence of giant cells in sepals.

We measured DNA content in the rosette leaves of *lgo-1* and wild type plants. Highly endoreduplicated 32C cells remain in *lgo* leaves (Figure S2E). Examining the leaves reveals that giant cells are absent similar to sepals corresponding with the reduction in 16C cells (Figure S2H–I); however, other endoreduplicated cell types such as hair cells (trichomes 32C) (Figure S2F–G) and the enlarged cells covering the midvein develop normally, which explains the presence of 32C cells in *lgo* leaves. *LGO* is expressed in trichomes so it is possible that the function of LGO in trichomes is obscured by redundancy with *SIAMESE* [62]. These results indicate that the function of *LGO* is developmentally specific to regulating endoreduplication in epidermal pavement cells to produce the cell size pattern. Similarly the *SIAMESE* gene, which is closely related to *LGO*, promotes endoreduplication specifically in trichomes [55].

### Altered Cell Cycle Times in *pATML1::KRP1* and *lgo-1* Shift the Cell Size Patterns

**Figure 6:**
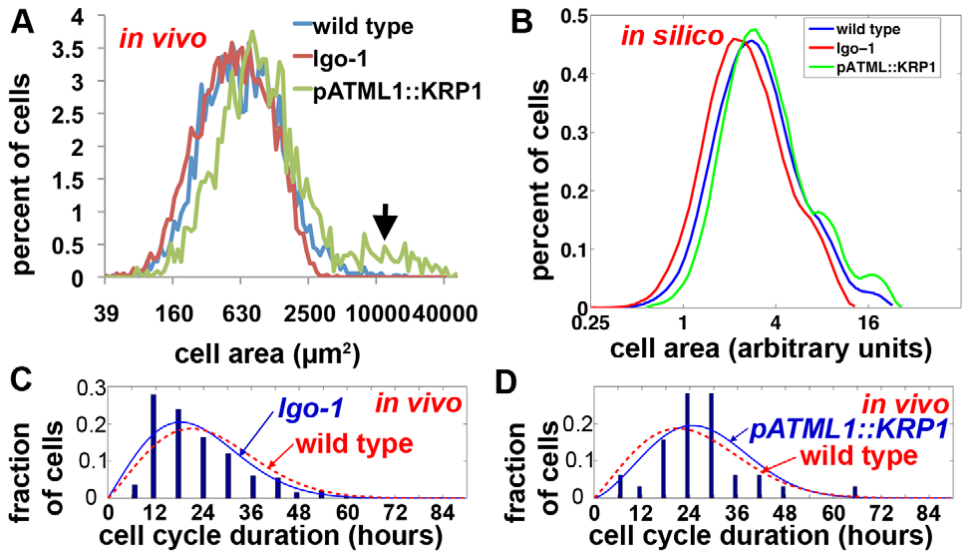
Changing the cell cycle duration shifts the resultant cell areas. (A) Comparison of the in vivo wild type (blue; reproduced from Figure 3J) cell size distribution to *lgo-1* (red) and *pATML1::KRP1* (green). Cell sizes were measured from images with semi-automated segmentation. The giant cells (black arrow) are lacking in *lgo-1* and increased in *pATML1::KRP1*. The overall size distribution is shifted toward larger cell sizes in *pATML1::KRP1* and slightly smaller cells in *lgo-1*. (B) *In silico* cell size distributions created by adjusting the endoreduplication probability and the cell cycle time distributions in the IG model to replicate *lgo-1* and *pATML1::KRP1*. Note the differences in the large cell peaks and the shift of the overall cell size curves are similar to the in vivo data. (C–D) In vivo histogram of the duration of the cell cycle in *lgo-1* (C) and *pATML1::KRP1* (D) sepal cells (dark blue bars) fit to a probability distribution (blue curve), showing a trend toward shorter (C) or longer (D) cell cycle times than wild type (red curve reproduced from Figure 3I). Note that in both cases the cell cycle times have 6-h resolution due to the time points in the live imaging.

We further tested whether the IGM model could reproduce the cell size distributions of *lgo-1* and *pATML1::KRP1*. We measured the in vivo cell size distributions of *lgo* and *pATML1::KRP1* using semi-automated image processing as we had for wild type sepals (Figures 6A and 3J). As expected, the largest cells corresponding to the giant cells are absent in *lgo-1* and increased in *pATML1::KRP1* (Figure 6A arrow). In addition, the entire cell size distribution curves are shifted slightly relative to wild type: *pATML1::KRP1* toward the right, indicating an overall increase in cell size, and *lgo* slightly toward the left, indicating a slight decrease in cell sizes. Measuring the cell cycle time distributions from the live imaging data showed that the average cell cycle time for *pATML1::KRP1* is longer than wild type and for *lgo-1* is shorter than wild type (Figure 6C–D; for statistics see Text S1). Sampling from these cell cycle time distributions as well as changing the probability of endoreduplication in the model produces cell size distributions reflecting both the effect on giant cells and the overall shifts in the cell size curves (Figure 6B). Again, this is an independent test of the model because the cell size data were not used to generate the model (see Text S1). The shifts in the cell size curves confirm that the length of the cell cycle and its regulation by cell cycle inhibitors is an important determinant of the cell size pattern.

### Endoreduplication Does Not Increase Overall Growth

While it is clear that endoreduplication promotes the enlargement of individual cells through lack of division, it is not clear whether endoreduplication increases the overall growth of the organ or tissue as has been proposed [7],[30],[40]. Contrary to this idea, the IGM model assumes that giant cells grow at the same rate per unit length as small cells, suggesting that changes in endoreduplication and the resulting cell size pattern will not affect the growth or resulting size of the sepal. We first tested the validity of this assumption by reexamining the live images of wild type sepals (Figure 2). The red giant cell and the entire neighboring brown cell lineage grow to the same size, supporting the assertion that growth is uniform in a local area and that cell size is controlled by the division of space created by the new cell walls (Figure 2C, yellow line). The endoreduplicated cell is not growing faster per unit wall length than the dividing cells.

Although growth of neighboring cells is uniform, growth on a global scale is not, as indicated by the smaller size of the developing giant cells in the bottom of the sepal than those at the top (Figure 2B compare red giant cell to the blue outlined giant cell). Understanding the global control of growth of the sepal is an open question for the future. The assumption of equivalent growth of neighboring cells is true on the local level and we retain it in the IGM.

**Figure 7:**
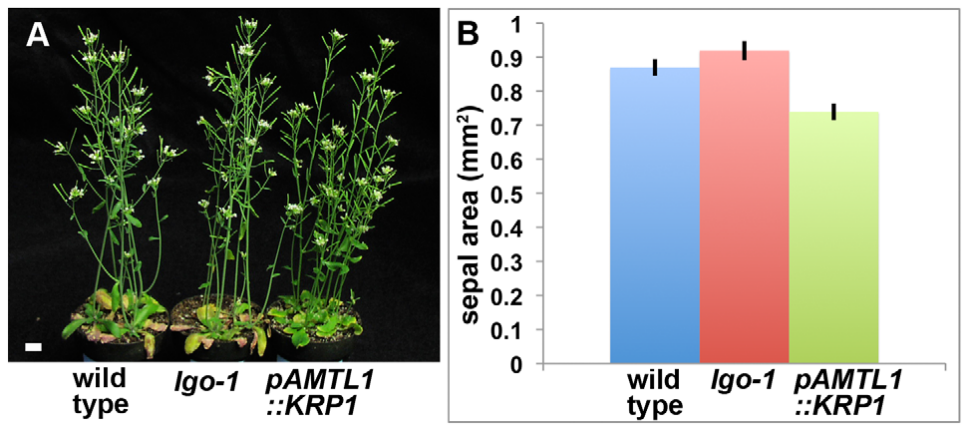
Endoreduplication does not increase overall growth of the organ. (A) Image of wild type, *lgo-1*, and *pATML1::KRP1* plants showing that they grow to approximately the same size. (B) Graph of the average areas of mature whole sepals showing the mutants have small effects on sepal area. Error bars represent the 95% confidence intervals. Scale bar: 1 cm.

In terms of the overall growth of the whole plant or the sepal, altering the proportion of cells endoreduplicating had little effect (Figure 7A–B). Contrary to expectation, increased endoreduplication in *pATML1::KRP1* plants has a slight inhibitory effect on growth (*p*<0.001), whereas decreased endoreduplication in *lgo* plants causes a slight increase in growth (*p*<0.5) (Figure 7B). These results demonstrate that endoreduplication does not increase the overall growth of the organ.

## Discussion

**Figure 8:**
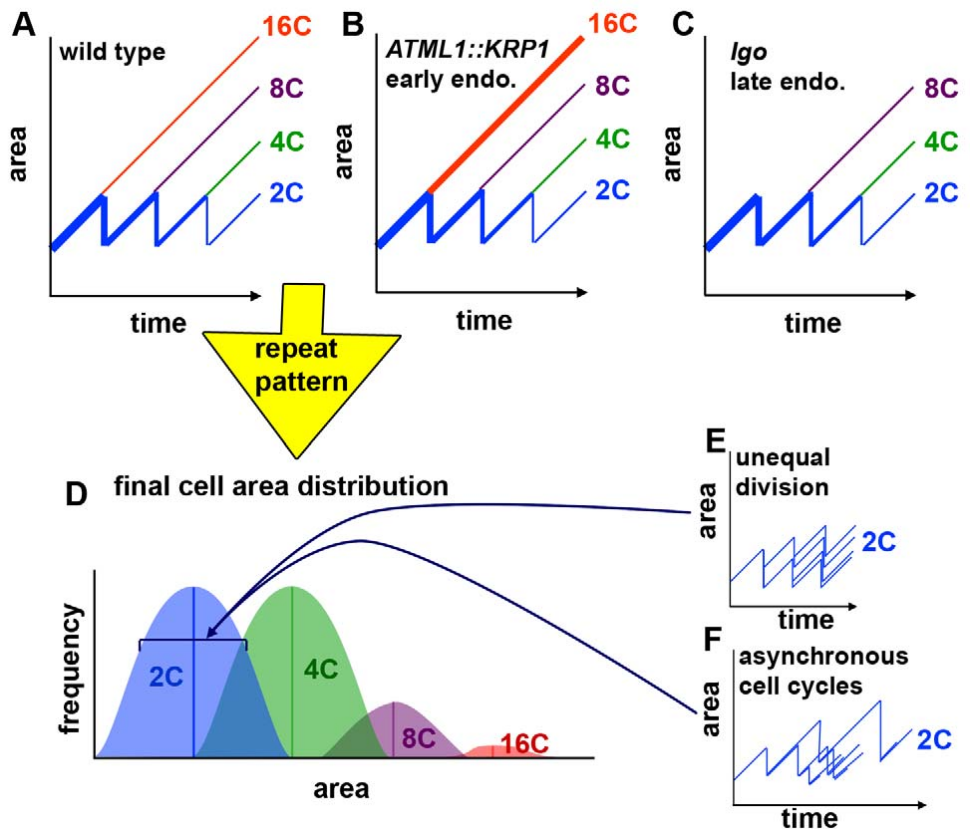
The timing of cell division creates the cell size pattern in the sepals. (A–C) Hypothetical graphs of the change in cell size over time in wild type (A), overexpression of the cell cycle inhibitor KRP1 (*pATML1::KRP1*) (B), and loss of cell cycle inhibitor activity in the *lgo* mutant (C). The size of 2C cells oscillates as they grow and divide. As cells decide to endoreduplicate, they exit the oscillating path and continue to grow throughout the remaining cell cycles. Their ultimate size depends on the cell cycle in which they started to endoreduplicate. Line weight roughly indicates the relative cell numbers. (D) Repeating the patterning cell cycles produces the cell size distribution of the sepal epidermis. The timing of endoreduplication creates the means for each ploidy as indicated by the lines. The range in cell sizes for each ploidy is created by unequal divisions (E) and asynchronous cell cycles (F). (E) Small differences in the sizes of daughter cells after a division add to variability in cell size. (F) The duration of cell cycles is variable. Throughout the cell cycle, the cells continue to grow, such that longer cycles result in larger cells, which add to the variability of cell sizes around the mean established by endoreduplication.

The computational model presented here is a step toward the development of a complete cellular model of the formation of a plant lateral organ. Combined with previous modeling of plant morphodynamics [20],[23],[24], it is a step closer to achieving a complete computational model of a plant. Our sepal model successfully reproduces the pattern of cell sizes in a developing sepal epidermis, showing that this pattern arises entirely from three parameters—the probability of a cell entering endoreduplication, variations in time of cell division, and variability in daughter cell size at division (Figure 8). Thus the simplest possible cellular information, acting on individual cells without any need for chemical messages sent between the cells, can explain the cell size pattern. Physical messages between cells, in the form of the attachment of cells to their neighbors through their shared cell walls, presumably are important, as this physical coupling of the cells must underlie their ability to grow at the same rates as their neighbors. Thus a combination of physical signaling and variable decisions is sufficient for the generation of a robust development pattern during flower development.

While cell size pattern is thus explained, the model has not yet been tested for the spatial distribution of cell sizes, which may require additional mechanisms. At some level the decision to become a giant cell is variable since the spatial pattern of giant cells changes from sepal to sepal. However, if a signaling network were to influence the decision to become a giant cell, a molecular network controlling LGO activity could be added to the model to bias the probability of becoming a giant cell depending on the signals received. Contrary to other patterning systems where specialized cells are spaced uniformly [63],[64], giant cells commonly form in clusters, suggesting the possibility of neighbor recruitment by endoreduplicating cells. KRP1 has been shown to act non-cell autonomously [43], and it is possible that intercellular movement of CDK inhibitors could coordinate neighboring cells.

Stochasticity is an important player in development [65],[66]. Random duration of the cell cycle has been shown to underlie the highly regular pattern of cells in the mouse ear [67]. Likewise, random noise in expression has been shown to be important in determining which *Bacillus subtilis* cells acquire the competence to take up DNA from the environment [68]. Models of many patterning systems rely on small random inhomogeneities in signaling molecules to initiate the pattern. For example, modeling the notch delta lateral inhibition patterning system is initiated with small fluctuations in the concentration of Notch [69]. Similarly, small fluctuations in the concentration of auxin in a model of the shoot apical meristem are sufficient to initiate the robust spiral pattern of primordia [13]. Here we show that there is variability in both the duration of the cell cycle and the decision to enter endoreduplication. In multicellular organisms the cell cycle generally becomes asynchronous after the initial cleavage divisions of the embryo when the cells start to grow [31], suggesting that cell cycle times are generally somewhat variable.

It is possible that the probability distribution in cell cycle length arises from noise in the underlying molecular mechanism. Key transitions in the cell cycle are regulated by active complexes of CYCLINs with CYCLIN DEPENDENT KINASES (CDKs); however, numerous factors regulate the formation of these complexes and their activity. One class of regulators includes the CDK inhibitors such as KRP1 and LGO that are thought to function by binding to and inactivating CYCLIN/CDK complexes [53],[70]. Specifically the KRP/ICK family of cell cycle inhibitors and the SIAMESE family have been shown to bind to CDKA;1 and CYCLIN D family members, which control G1 to S transition in the cell cycle [53],[54],[58],[59]. KRP1 is regulated both transcriptionally and post-translationally through regulation of nuclear localization and through proteasomal degradation [59],[71]. *LGO* is transcriptionally repressed by the growth promoting plant hormone gibberellin [72] and is upregulated by certain types of stress [54].

Our results show that CDK inhibitors affect both the distribution of cell cycle times and the probability of endoreduplication. Given that CDK inhibitors serve to inhibit transitions in the cell cycle, our results can be interpreted to indicate that increasing this inhibition through overexpression of *KRP1* causes a shift toward longer cell cycles, whereas removing this inhibition in *lgo-1* mutants causes faster cell cycles, which consequently results in larger or smaller cell sizes, respectively (Figure 6). Likewise CDK inhibitors have been shown to promote endoreduplication in both plants and animals [52],[70],[73]. Medium levels of *KRP2* overexpression trigger endoreduplication, while high levels of *KRP2* overexpression completely block the cell cycle [52]. The *pATML1::KRP1* overexpression lines have medium levels of expression [51] and thus trigger endoreduplication. However, as we see, the promoting of endoreduplication is probabilistic because some cells escape endoreduplication to make the islands of small cells in *pATML1::KRP1* despite the expression of *KRP1* throughout the epidermis. In fact, the number of endoreduplicating cells is variable and on average merely doubled in *pATML1::KRP1* sepals; however, the area of those cells is enlarged due to the longer cell cycle such that they take up a disproportionate area of the sepal surface and therefore appear predominant (Figure 4C–D).

Our results from the live imaging of sepal development reveal many parallels between leaf and sepal growth. First, in leaves it has been shown that division ceases in a basipetal wave from the top downward similar to what we see in the sepal [47],[59]. Second, the growth and division patterns are similar as indicated by the resemblance of the shape of the patch of cells belonging to a lineage in the sepal (the equivalent of a sector) to the sectors generated in developing leaves [74]. Third, both leaves and sepals have giant cells [7],[47]. The main differences between the sepal and the leaf blade are that pavement cells on leaves are lobed, leaves reach a larger size, and leaves have more trichomes. Our analysis is providing insight into the universal principles of plant organ growth, much in the spirit of Goethe's suggestion in 1790 that floral organs are merely modified leaves [75].

Plant organs show a remarkable ability to reach the correct size and shape despite perturbations to their cell division patterns. For example, in *tangled-1* maize mutants, leaf shape is preserved despite the aberrant orientations of many divisions [76]. Likewise, when an organ has fewer cells, the cells often compensate by increasing their expansion such that the organ formed approaches normal size [77]. Compensation has been shown to occur during different phases of growth in different mutants [78]. Plants overexpressing the cell cycle inhibitor KRP2 have been shown to undergo compensation during the proliferation phase; however, the mechanism of compensation is unknown [78]. Here we also observe near conservation of sepal size despite differences in the numbers of cells in wild type, *lgo*, and *pATML1::KRP1*, indicating that the sepals are undergoing compensation (Figures 7B and S2J). We show that *pATML1::KRP1* fits the compensation paradigm of having fewer cells, which are larger through the mechanism of both an increase in ploidy as well as an increase in the cell cycle time. Conversely, the *lgo-1* sepals have more cells, which are smaller due to both a decrease in endoreduplication and an average decrease in the duration of the cell cycle. In both these cases, compensation does not occur due to any fundamentally new mechanism but is an outcome of extending the variability of the wild type cell cycle distribution to higher or lower values. Future use of live imaging and modeling should be able to further unravel the complexities of the feedback loops that exist between cell cycle and growth. The mechanism that determines the ultimate size of a plant organ remains an active area of research, and insight is likely to be gained through future application of these strategies to mutants affecting organ size such as *big brother*, *aintegumenta*, and *jagged* [79]–[82]. In *Drosophila* wing disk development, morphogens and mechanics play a role in controlling organ size [18].

Currently the function of having a wide range of pavement cell sizes is unclear [2]. The prevalence of the cell size pattern throughout the epidermis of most *Arabidopsis* organs [7],[47] suggests the patterning might have a function in many locations. It is possible that the diversity in cell sizes plays a role in defense against insect predators, helps the plant respond to water stress, or has a mechanical role. Of these three, the mechanical role seems plausible because the elastic properties of a large cell filled with water would be different from those of several smaller cells. Mechanics could be involved in sepal development because the sepal curves to cover the developing flower in immature stages but later opens, allowing the flower to bloom at maturity. Overexpression of the cell cycle inhibitor *KRP1* in the epidermis of the *pATML1::KRP1* plants causes the sepals to curve outward, opening the flower early and exposing the developing organs (Figure 4B) [51]. However, it is not clear whether this dramatic change in curvature is due to the increased production of giant cells or some other effect of *KRP1* overexpression. Conversely *lgo* sepals tend to remain curved inward after wild type sepals flatten. Future tests are required to determine how cell size affects mechanical properties of tissues.

Understanding plant growth is important for the cellulosic biofuels industry, which focuses on harvesting cellulose from the cell walls [83]. The *lgo* mutant has more cell walls within the same space as wild type plants and consequently would be expected to have more cellulose, although this remains to be tested. The relative normality of *lgo* mutants in our growth conditions despite the production of these extra cell walls suggests that generally decreasing cell size through additional cell divisions may be useful in the production of cellulose feedstocks.

Here we have shown the sufficiency of variable cell division decisions in the model to generate the observed dynamics of the distribution of cell sizes. We have also shown that a combination of live imaging and successive iterations of computational models, altered by addition of newly acquired developmental information, can bring us progressively closer to detailed and predictive models of plant growth and to uncovering the basic principles of plant organ growth.

## Materials and Methods

### Computational Image Analysis

Computational programs (described in Text S1) were used to measure sepal areas, sepal epidermal cell areas, and quantify the number of cells in the sepal through counting nuclei.

### Live Imaging of Sepal Development

Live imaging was conducted according to procedures in [10],[84], except that the experimental details of plant growth and manipulation were altered to observe the lateral side of sepals (for details, see Text S1). Briefly, plants expressing pAR98 *pATML1::H2B-mYFP* were grown in pots, the stems were taped to slides, the overlying flowers were dissected away, and the inflorescence with young sepals were mounted under a cover slip, stained with propidium iodide (PI), and imaged on a Zeiss 510 meta confocal laser scanning microscope every 6 (or 12) h. The resulting images were cropped in Amira 4.1 software (Visage Imaging, Mercury Computer Systems) to remove neighboring flowers, registered, and snapshots of volume renderings were exported (Figure S4). Cell lineages were manually tracked in Adobe Photoshop CS as in [10],[11] (Figure S4; for additional details, see Text S1).

To determine the change in cell area over time, live imaging was performed on sepals where both the nuclei pAR98 (*pATML1::H2B-mYFP*) and plasma membranes pAR169 (*pATML1::mCitrine-RCI2A*) in the epidermis were visualized. The cells in individual lineages were measured at each time point using ImageJ 1.41o (http://rsbweb.nih.gov/ij/).

### Computational Modeling

Models were created in Matlab. For details see Text S1.

### DNA Quantification

Flow cytometry was performed as described [85] using a FACSCalibur four color analyzer. 50 stage 12 sepals or 6 rosette leaves were used in each sample. To distinguish the ploidy of the epidermal cells from the internal cells in the sepal, the epidermal nuclei were labeled with GFP (pAR180 and pAR181 *pATML1::H2B-mGFP*). Nuclei from whole sepals of transgenic plants were prepared and stained with PI as described [85]. Additional gating was used to separate the GFP positive epidermal nuclei from the GFP negative internal nuclei. Histograms of the PI fluoresce for each population showed the relative DNA content of each population.

DNA and cell area were quantified from DAPI stained sepals prepared as described [86] and imaged with 780 nm two-photon excitation on a Zeiss LSM 510 Meta NLO microscope with Coherent Chameleon. Image J was used to measure both the integrated density of the nucleus and a corresponding region for background subtraction from a single Z-section and the area of the cell from a different Z-section.

### Accession Numbers

The *Arabidopsis* genome initiative numbers of genes mentioned in this study are: *ATML1* (At4g21750); *H2B* (At5g22880); *KRP1* (At2g23430); *LGO* (At3g10525); *RCI2A* (At3g05880); and *SIM* (At5g04470).

## Supporting Information

Figure S1 **Asynchronous cell cycles and unequal divisions contribute to diversity in cell size.** Related to Figure 2. (A and C) Time series data are shown for the brown (A) and green (C) small cell lineages from Video S2. Daughter cells resulting from a division are circled in white. Divisions that form meristemoids are labeled M and guard cells labeled g. Endoreduplicating cells are marked with an E. (B and D) Graphs showing the areas of the cells in the brown lineage (B) and the green lineage (D) over time. Normal divisions are marked with an asterisk, divisions to form a meristemoid in the stomatal lineage are marked with an M, and divisions that form guard cells are marked with a g [39]. The final endoreduplicating cells are marked with an E and guard cells are marked with a g. Scale bars: 10 µm.
(1.67 MB TIF)

Figure S2 ***lgo*** **mutants block giant cell formation, but not endoreduplication.** Related to Figure 4. (A, B) SEMs of a wild type mature stage 14 sepal (Columbia accession). Giant cells are false colored red. (C, D) SEMs of a *lgo-2* stage 14 sepal (Columbia accession) showing the absence of giant cells, but a range of small cell sizes. (E) Graph of the average percent ploidy of rosette leaves for wild type Landsberg (Ler), *lgo-1* (in the Ler background), *pATML1::KRP1* (in the Ler background), wild type Columbia (Col), and *lgo-2* (in the Col background). In both *lgo* alleles the number of 4C cells is increased and the number of 16C and 32C cells is decreased but still present. 32C cells are shown in the inset. The SIAMESE family cell cycle inhibitors are thought to function at the G1 to S transition, so it would be expected that that progression through this stage of the cell cycle would be faster resulting in more 4C cells in G2 [53],[60]. (F) Wild type Ler rosette leaf trichome. (G) *lgo-1* rosette leaf trichome, which is normal shape and size. (H) The wild type abaxial leaf epidermis also contains a range of cell sizes from giant cells (false colored red) to small cells. (I) The *lgo-1* abaxial leaf epidermis lacks giant cells. (J) The number of cells in the entire sepal epidermis was quantified from images of fluorescently tagged histones using two segmentation methods (see procedures). The graph shows that wild type (Ler) sepals have about 1,600 cells in the front (abaxial) epidermis and about 2,800 in the back (adaxial) epidermis. These numbers were used as parameters in the computational models. The number of cells in the *lgo-1* abaxial epidermis is increased as expected because multiple small cells replace giant cells. Conversely, *pATML1::KRP1* sepals have fewer cells in the abaxial epidermis because the additional cells entering the giant cell pathway have no progeny. Scale bars: 100 µm.
(3.96 MB TIF)

Figure S3 **Overexpression of** ***LGO*** **produces ectopic giant cells.** Nuclei (*pATML1::H2B-mYFP*) are shown in green and cell walls (PI) are shown in red. Giant cells have large nuclei and large area. Round red stained cells are guard cell pairs. (A) Wild type stage 12 mature sepal showing the normal proportion of giant cells. (B) *pATML1::KRP1* sepals have approximately double the number of giant cells as wild type. (C) *pATML1::LGO* sepals are similar to *pATML1::KRP1* sepals. Scale bar: 100 µm.
(4.41 MB TIF)

Figure S4 **Methodology for tracking cell lineages.** (A–B) Projection images formed from confocal stacks before any processing at the second time point (A) and third time point (B). Nuclear (*pATML1::H2B-mYFP*) fluorescence is shown in green and cell wall staining (PI) in red. (C–D) The 3D confocal stack images were cropped in 3D using the volume edit function in Amira to remove parts of other flowers in the field of view. This was necessary for the subsequent alignment of the images using the affine registration function in Amira. The images were visualized in 3D using the Voltex volume rendering function in Amira and snapshots were taken of all time points at the same magnification and the same angle. Nuclei are shown in gold and cell walls in green. (E–F) Colored lineage dots are transferred from their known position in the earlier time point (E) to the later time point (F). For reference, the daughter nuclei resulting from a division (as determined in H) are outlined in white. (G) In time point 3, the lineage dots that match cells, which have not divided, are moved to overlie the position of the same nucleus. Movement is required due to the growth of the sepal. It is easiest to determine which nuclei have divided and which have not by flipping between consecutive time points in a video. (H) Generally those nuclei that divide will have one colored dot near two new nuclei. This dot is duplicated and positioned over each new nucleus. The new nuclei are also circled in white to indicate the result of a division event. Scale bars: 10 µm.
(4.67 MB TIF)

Text S1 **Supplemental Procedures and Modeling Supplement.**
(1.65 MB PDF)

Video S1 **Live imaging of wild type sepal primordium development.** Related to Figure 2. A wild type sepal primordium was imaged every 6 h for 72 h. Corresponding images are displayed in Figure 2A–C.
(0.37 MB MOV)

Video S2 **Live imaging of wild type sepal primoridum development.** Related to Figure 2. A wild type sepal primordium was imaged every 6 h for 72 h. Both the nuclei (*pATML1::H2B-mYFP*) and the plasma membranes (*pATML1::mCitrine-RCI2A*) of the epidermal cells are shown in gold. Note that the division immediately preceding giant cell formation is captured in the red and blue lineages on the left. The cells in this video form the basis for area measurements in Figures 2D and S1.
(0.38 MB MOV)

Video S3 **Live imaging of wild type sepal primoridum development.** Related to Figure 2. A wild type sepal primordium was imaged every 6 h for 102 h. Please ignore the black line in the center of the images because it is an artifact of image export. Note that the copper-rose colored nucleus in the middle right part of the sepal shows the rare exception of an endoreduplicating cell that divides.
(0.51 MB MOV)

Video S4 **Intercalary Growth model showing early sepal development.** Related to Figure 3. The *in silico* sepal develops from a generative layer that continues to proliferate throughout development. As cells exit the generative layer, they enter patterning divisions, in which they can divide or endoreduplicated. The ploidy of cells is indicated by their color: blue, 2C; green, 4C; magenta, 8C; and red, 16C. Corresponding images are displayed in Figure 3G.
(0.47 MB MPG)

Video S5 **Live imaging of the formation of the wild sepal primoridum.** Related to Figure 3. A wild type stage 2 floral meristem was imaged every 6 h for 60 h to show the emergence of the sepal. Corresponding images are displayed in Figure 3A–B. Note that the bright stop appearing on the top of the sepal in frame 6 is a pollen grain that has fallen on the sepal and germinated.
(0.31 MB MOV)

Video S6 **Live imaging of the apical to basal wave in the termination of cell division in wild sepal development.** Related to Figure 3. A developing wild type lateral sepal was imaged every 6 h for 54 h. Both the nuclei (*pATML1::H2B-mYFP*) and the plasma membranes (*pATML1::mCitrine-RCI2A*) of the epidermal cells are shown in gold. Only the cell divisions are indicated with white outlines of daughter nuclei. The vertical location of these cell divisions is plotted in Figure 3D and the 54-h time point is shown in Figure 3C.
(2.94 MB MOV)

Video S7 **Ectopic giant cells computational model.** Related to Figure 4. Increasing the probability of endoreduplication in the first cell cycle (p_1_) produces sepals *in silico* with extra giant cells similar to the phenotype of plants ectopically expressing the cell cycle inhibitor *pATML1::KRP1*.
(0.42 MB MPG)

Video S8 **Loss of giant cells computational model.** Related to Figure 4. Decreasing the probability of entering endoreduplication in the first cell cycle (p_1_ = 0) produces sepals *in silico* without giant cells similar to the phenotype of *lgo-1* mutants, which have lost the activity of the LGO cell cycle inhibitor.
(0.49 MB MPG)

Video S9 **Live imaging of** ***pATML1::KRP1*** **sepal primoridum development.** Related to Figure 5. A *pATML1::KRP1* sepal primordium was imaged every 6 h for 60 h. Corresponding images are displayed in Figure 5A–C. Note that most of the cells endoreduplicate. Only two divisions of the tracked cells occur during the sequence and both form guard cells.
(0.23 MB MOV)

Video S10 **Live imaging of** ***pATML1::KRP1*** **sepal primoridum development.** Related to Figure 5. A *pATML1::KRP1* sepal primordium was imaged every 6 h for 72 h.
(0.39 MB MOV)

Video S11 **Live imaging of** ***pATML1::KRP1*** **sepal primoridum development.** Related to Figure 5. A *pATML1::KRP1* sepal primordium was imaged every 6 h for 84 h.
(0.30 MB MOV)

Video S12 **Live imaging of** ***lgo-1*** **sepal primoridum development.** Related to Figure 5. A *lgo-1* sepal primordium was imaged every 6 h for 72 h. Corresponding images are displayed in Figure 5E–G. Note that this time lapse starts with a younger sepal primordium than other videos. The 12-h time point better corresponds to the starting point of the other videos.
(0.19 MB MOV)

Video S13 **Live imaging of** ***lgo-1*** **sepal primoridum development.** Related to Figure 5. A *lgo-1* sepal primordium was imaged every 6 h for 102 h.
(0.52 MB MOV)

Video S14 **Live imaging of** ***lgo-1*** **sepal primoridum development.** Related to Figure 5. A *lgo-1* sepal primordium was imaged every 6 h for 78 h.
(0.39 MB MOV)

## Abbreviations

ATML1: *Arabidopsis thaliana* MERISTEM LAYER 1
CDK: CYCLIN DEPENDENT KINASE
H2B: HISTONE 2B
IGM: Intercalary Growth Model
KRP1: KIP RELATED PROTEIN1
LGO: LOSS OF GIANT CELLS FROM ORGANS
PI: propidium iodide
