# Supplementary figures and images for: Variability in the Control of Cell Division Underlies Sepal Epidermal Patterning in Arabidopsis thaliana
Source: PLoS Biol. 2010 May 11;8(5):e1000367. doi: 10.1371/journal.pbio.1000367 (PMC2867943; doi:10.1371/journal.pbio.1000367)

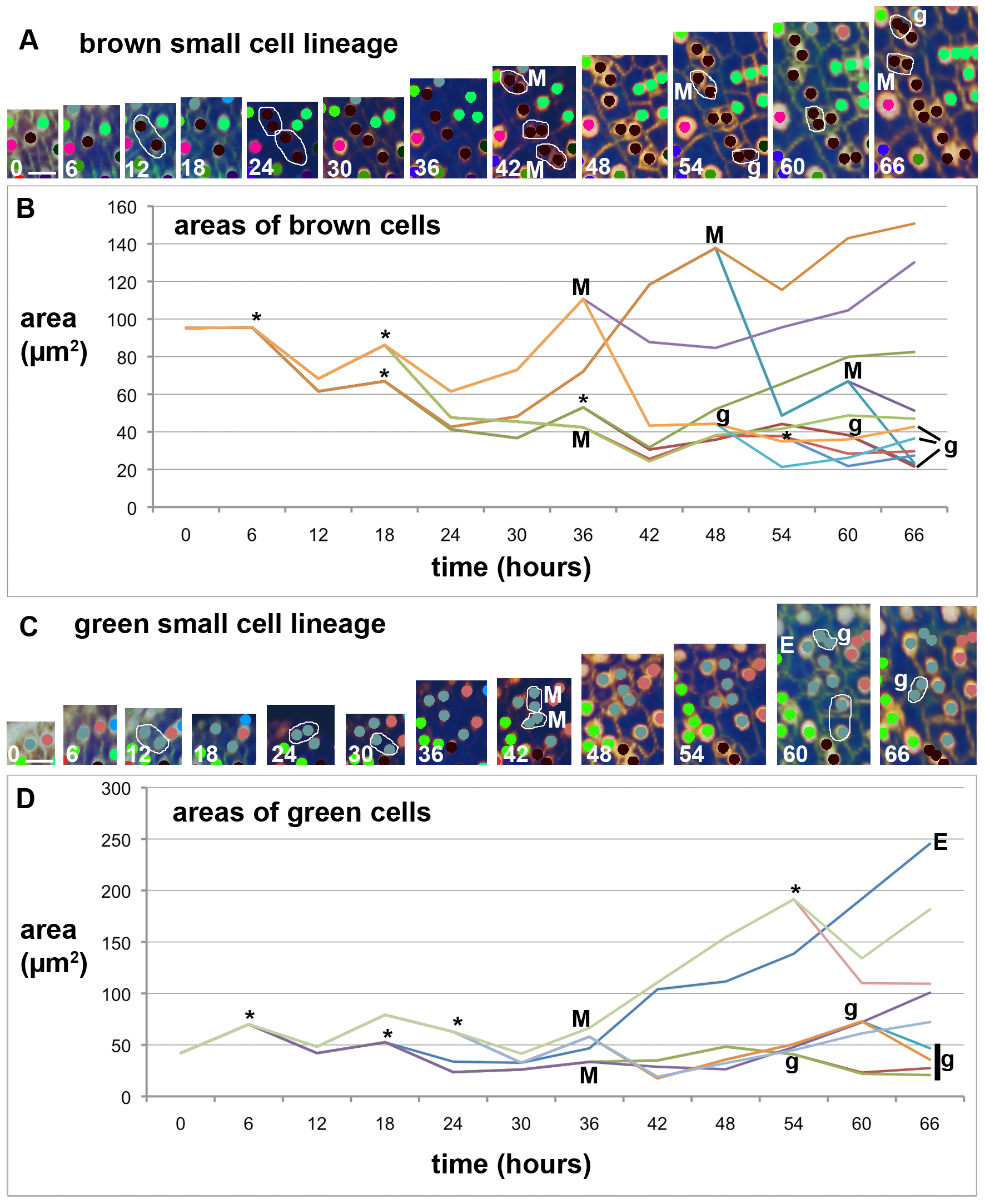

Supplement: Figure S1 — Asynchronous cell cycles and unequal divisions contribute to diversity in cell size. Related to Figure 2. (A and C) Time series data are shown for the brown (A) and green (C) small cell lineages from Video S2. Daughter cells resulting from a division are circled in white. Divisions that form meristemoids are labeled M and guard cells labeled g. Endoreduplicating cells are marked with an E. (B and D) Graphs showing the areas of the cells in the brown lineage (B) and the green lineage (D) over time. Normal divisions are marked with an asterisk, divisions to form a meristemoid in the stomatal lineage are marked with an M, and divisions that form guard cells are marked with a g [39]. The final endoreduplicating cells are marked with an E and guard cells are marked with a g. Scale bars: 10 µm. (1.67 MB TIF) [file pbio.1000367.s001.tif]

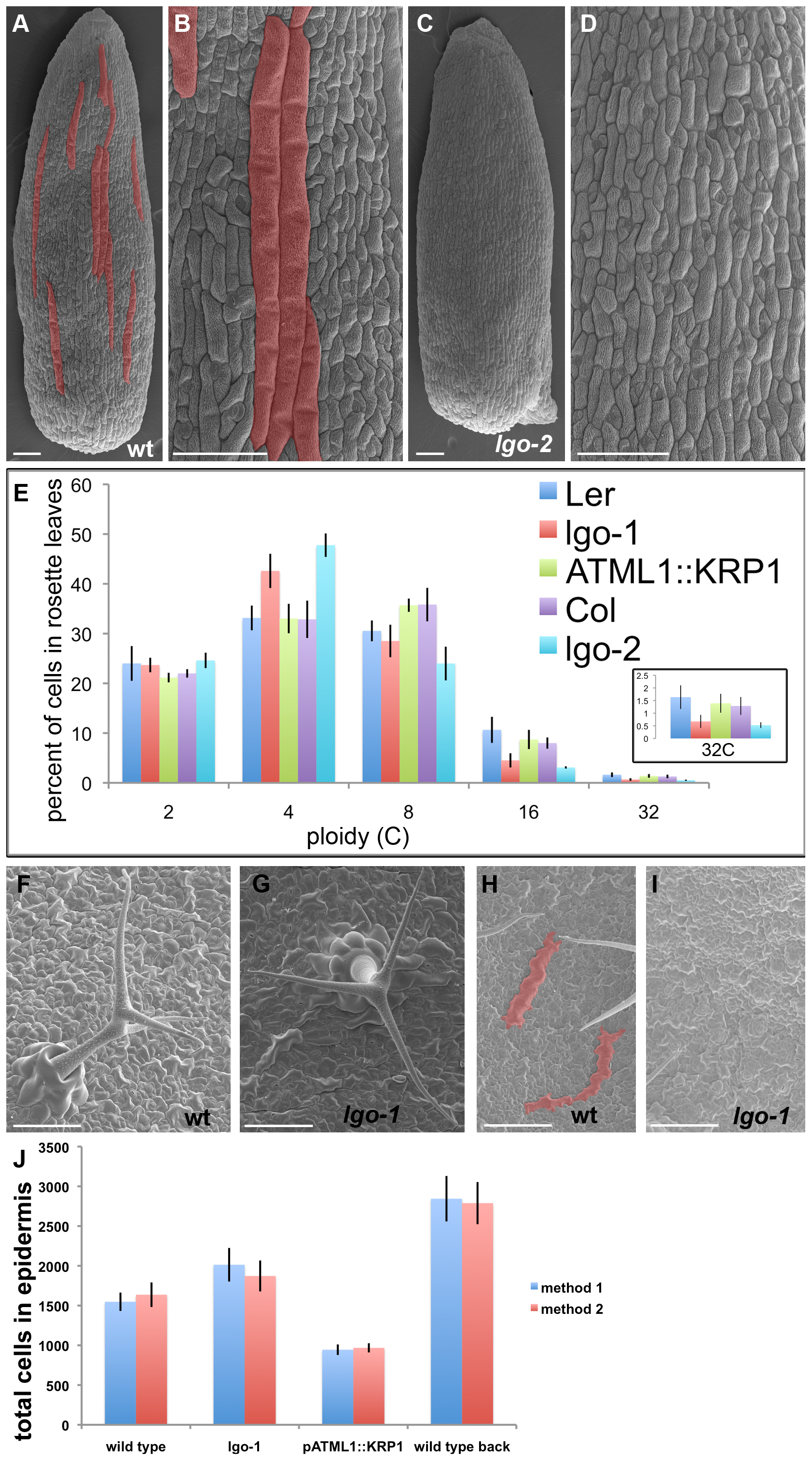

Supplement: Figure S2 — lgo mutants block giant cell formation, but not endoreduplication. Related to Figure 4. (A, B) SEMs of a wild type mature stage 14 sepal (Columbia accession). Giant cells are false colored red. (C, D) SEMs of a lgo-2 stage 14 sepal (Columbia accession) showing the absence of giant cells, but a range of small cell sizes. (E) Graph of the average percent ploidy of rosette leaves for wild type Landsberg (Ler), lgo-1 (in the Ler background), pATML1::KRP1 (in the Ler background), wild type Columbia (Col), and lgo-2 (in the Col background). In both lgo alleles the number of 4C cells is increased and the number of 16C and 32C cells is decreased but still present. 32C cells are shown in the inset. The SIAMESE family cell cycle inhibitors are thought to function at the G1 to S transition, so it would be expected that that progression through this stage of the cell cycle would be faster resulting in more 4C cells in G2 [53],[60]. (F) Wild type Ler rosette leaf trichome. (G) lgo-1 rosette leaf trichome, which is normal shape and size. (H) The wild type abaxial leaf epidermis also contains a range of cell sizes from giant cells (false colored red) to small cells. (I) The lgo-1 abaxial leaf epidermis lacks giant cells. (J) The number of cells in the entire sepal epidermis was quantified from images of fluorescently tagged histones using two segmentation methods (see procedures). The graph shows that wild type (Ler) sepals have about 1,600 cells in the front (abaxial) epidermis and about 2,800 in the back (adaxial) epidermis. These numbers were used as parameters in the computational models. The number of cells in the lgo-1 abaxial epidermis is increased as expected because multiple small cells replace giant cells. Conversely, pATML1::KRP1 sepals have fewer cells in the abaxial epidermis because the additional cells entering the giant cell pathway have no progeny. Scale bars: 100 µm. (3.96 MB TIF) [file pbio.1000367.s002.tif]

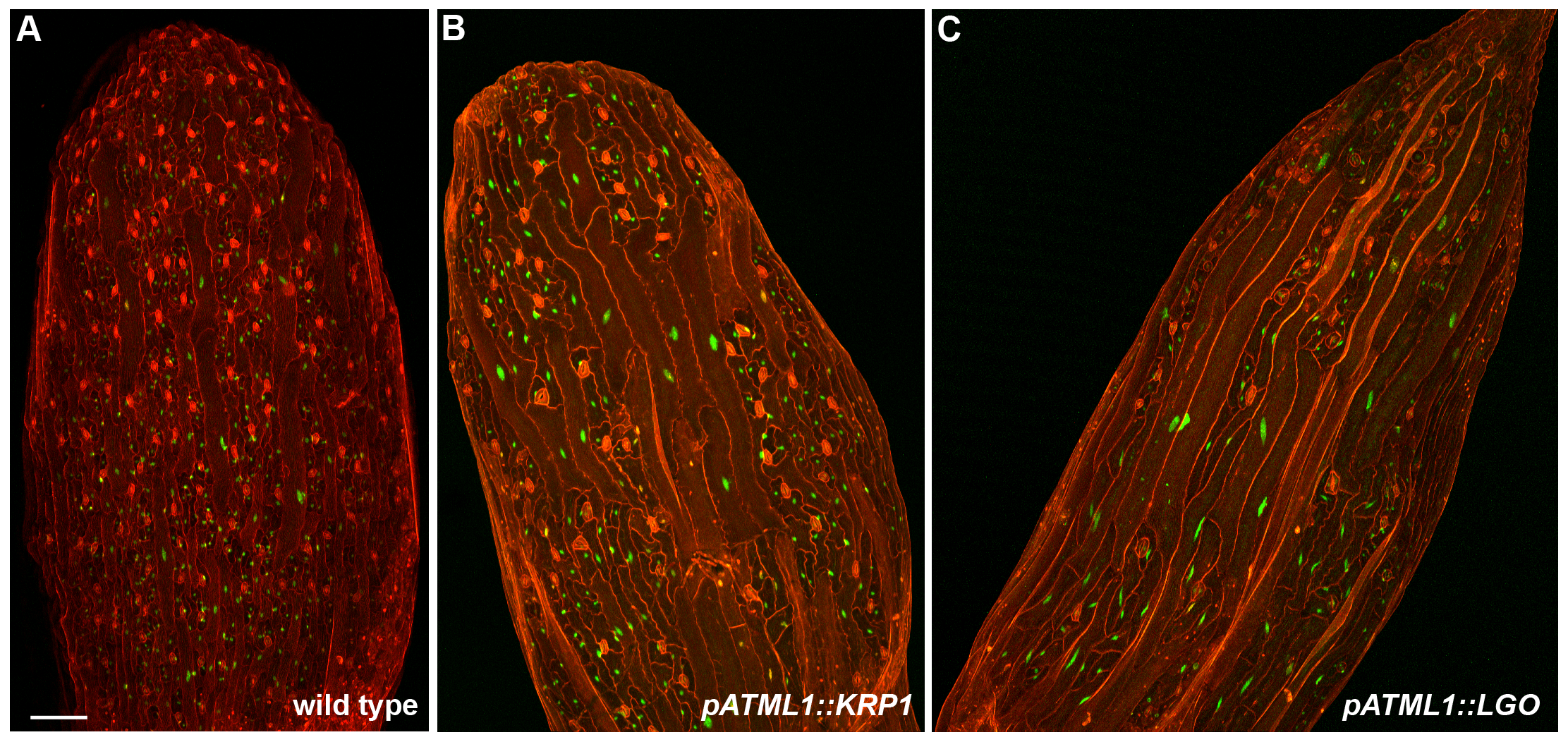

Supplement: Figure S3 — Overexpression of LGO produces ectopic giant cells. Nuclei (pATML1::H2B-mYFP) are shown in green and cell walls (PI) are shown in red. Giant cells have large nuclei and large area. Round red stained cells are guard cell pairs. (A) Wild type stage 12 mature sepal showing the normal proportion of giant cells. (B) pATML1::KRP1 sepals have approximately double the number of giant cells as wild type. (C) pATML1::LGO sepals are similar to pATML1::KRP1 sepals. Scale bar: 100 µm. (4.41 MB TIF) [file pbio.1000367.s003.tif]

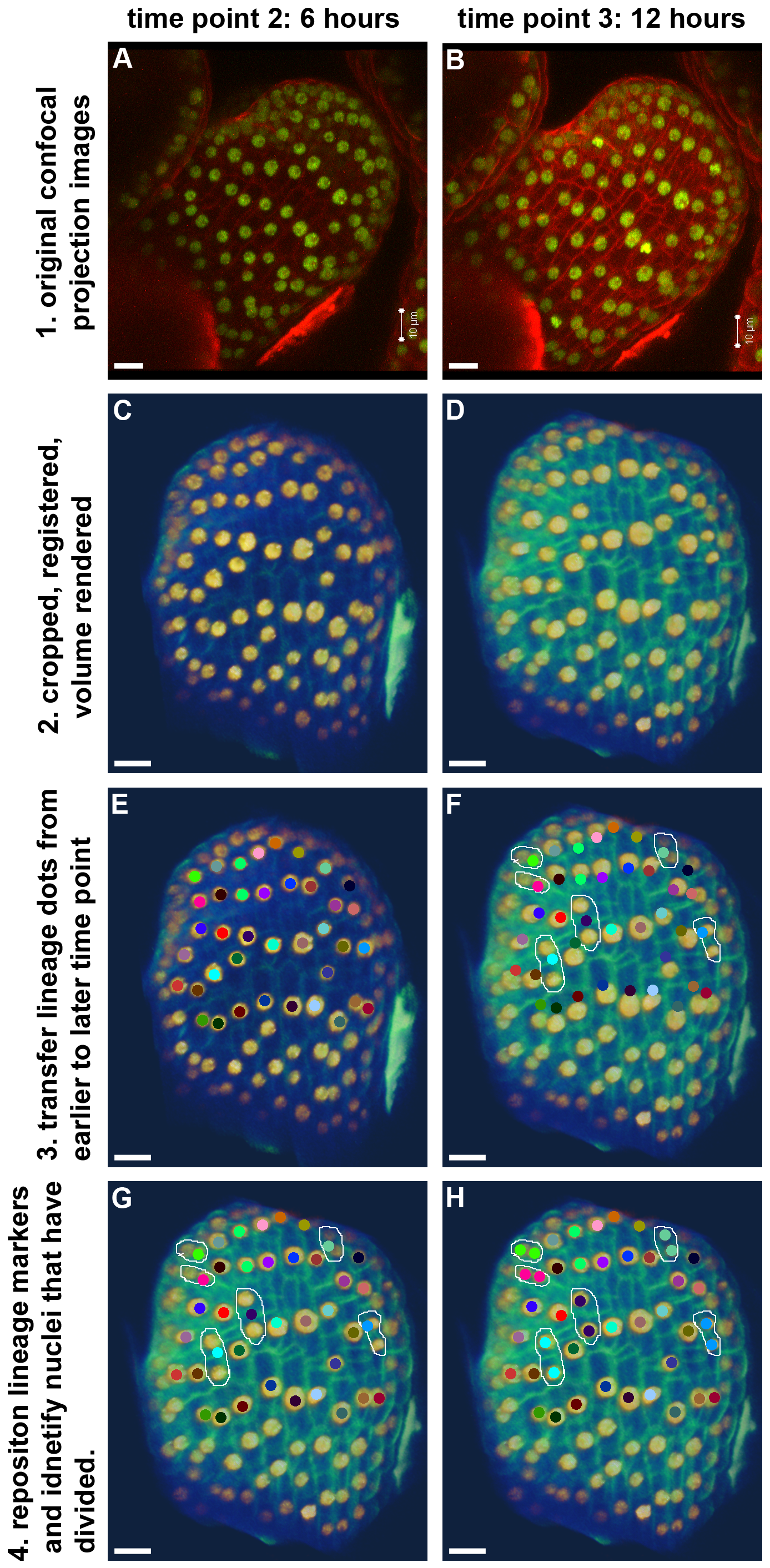

Supplement: Figure S4 — Methodology for tracking cell lineages. (A–B) Projection images formed from confocal stacks before any processing at the second time point (A) and third time point (B). Nuclear (pATML1::H2B-mYFP) fluorescence is shown in green and cell wall staining (PI) in red. (C–D) The 3D confocal stack images were cropped in 3D using the volume edit function in Amira to remove parts of other flowers in the field of view. This was necessary for the subsequent alignment of the images using the affine registration function in Amira. The images were visualized in 3D using the Voltex volume rendering function in Amira and snapshots were taken of all time points at the same magnification and the same angle. Nuclei are shown in gold and cell walls in green. (E–F) Colored lineage dots are transferred from their known position in the earlier time point (E) to the later time point (F). For reference, the daughter nuclei resulting from a division (as determined in H) are outlined in white. (G) In time point 3, the lineage dots that match cells, which have not divided, are moved to overlie the position of the same nucleus. Movement is required due to the growth of the sepal. It is easiest to determine which nuclei have divided and which have not by flipping between consecutive time points in a video. (H) Generally those nuclei that divide will have one colored dot near two new nuclei. This dot is duplicated and positioned over each new nucleus. The new nuclei are also circled in white to indicate the result of a division event. Scale bars: 10 µm. (4.67 MB TIF) [file pbio.1000367.s004.tif]
