# Supplementary material for: Variability in the Control of Cell Division Underlies Sepal Epidermal Patterning in Arabidopsis thaliana
Source: PLoS Biol. 2010 May 11;8(5):e1000367. doi: 10.1371/journal.pbio.1000367 (PMC2867943; doi:10.1371/journal.pbio.1000367)
Supplement: Text S1 — Supplemental Procedures and Modeling Supplement. (1.65 MB PDF) [file pbio.1000367.s005.pdf]

Supplemental Data for

Variability in the Control of Cell Division Underlies Sepal Epidermal Patterning in *Arabidopsis thaliana*

Adrienne H. K. Roeder, Vijay Chickarmane, Alexandre Cunha, Boguslaw Obara, B. S. Manjunath, Elliot M. Meyerowitz

Supplemental Procedures

Image processing

Modeling Supplement

Supplemental Procedures:

Isolation and positional cloning of the *LGO* gene

The *lgo-1* mutant was isolated in a screen to identify mutants with defects in giant cells. The sepals of M2 families of Landsberg erecta (Ler) wild type *Arabidopsis thaliana* plants that had been mutagenized with ethyl methane sulfonate (EMS) (purchased from Lehle Seeds) were observed with a dissecting microscope.

The *lgo-1* mutation (in Ler accession) was mapped in the F2 of a cross to a wild type Columbia (Col) accession plant. 91 homozygous mutants were selected from the segregating population and the mutation was mapped to between the polymorphic markers nga126 and nga162 on Chromosome 3 [1]. The *SIAMESE RELATED1* gene At3g10525 is located within this interval and sequencing showed that the *lgo-1* mutation is a C to T base pair change at base 184 of the coding sequence (Figure 4J). A second *lgo-2* allele (SALK_033905) caused by a T-DNA insertion after base 294 confirms that mutations in the *LGO* gene cause the loss of giant cells phenotype (Figure S2A-D).

Genotyping of *lgo*

The *lgo-1* mutation can be PCR genotyped by amplifying with oAR296 (5'-TGGTGGCCGGGGTTTTCTCA-3') and oAR297 (5'-CAAAGAAGGACGAAGGTGATG-3') followed by digesting the product with DdeI to produce a 104 bp wild type product or a 125 bp mutant product. The *lgo-2* allele can be PCR genotyped by amplifying with LGO specific flanking primers oAR284 (5'-CTTCCCTCTCACTTCTCCAA-3') and oAR285 (5'-CCGAACACCAACAGATAATT-3') as well as T-DNA specific primer JMLB2 (5'-

TTGGGTGATGGTTCACGTAGTGGG-3') to generate a 546 bp wild type product or a 753 bp mutant product.

Transgenic plants

The *pATML1::KRP1* plants line 16-3 in Landsberg erecta (Ler) were kindly provided by Dr. Keiko Torii [2].

The *pATML1::H2B-mYFP* construct was made by replacing the GUS gene from pBI101 (Clonetech) with the Hind III to Sac I linker from pGEM7Z (Promega) to form plasmid pAR94. The *ATML1* promoter fragment was excised from pAS99 [3] with Hind III and inserted into the Hind III site of pAR94 to create an epidermal expression vector pAR95. The DNA encoding the Histone2B-mYFP fusion protein was excised from *35S::H2B-mYFP* [4] with Bam HI and Sac I and inserted into pAR95 to create pAR98 (*pATML1::H2B-mYFP*). pAR98 was electroporated into *Agrobacterium* and transformed into Ler plants by floral dipping. Transgenic plants were selected for resistance to Kanamycin.

Two versions of *pATML1::H2B-mGFP* were generated with different selectable markers. The sequence of eGFP was modified (A206K) to be monomeric (mGFP). The Histone2B gene was amplified with primers oAR369 (5'-CACCGGATCCACAATGGCGAAGGCAGATAAG-3') and oAR368 (5'-AGCGGCAGCAGCCGCAGCAGGAGAACTCGTAAACTTCGTAAC-3') from 35S::H2B-mYFP [4] and mGFP amplified from mGFP-TOPO with oAR367 (5'-GTTACGAAGTTTACGAGTTCTCCTGCTGCGGCTGCTGCCGCT-3') and oAR366 (5'-gggagctcTTACTTGTACAGCTCGTCCATGCC-3'). Histone 2B was fused to GFP through PCR with oAR369 and oAR366 using both the H2B and GFP PCR products as templates. The product was cloned into pENTR/D-TOPO (Invitrogen) to generate the pAR179 H2B-mGFP

entry clone. H2B-mGFP was excised from pAR179 with Bam HI and Sac I and cloned into pAR95 to created pAR181 *pATML1::H2B-mGFP* with Kanamycin resistance in plants. A gateway epidermal specific expression vector pAR176 was generated by replacing the 35S promoter from pB7WG2.0 [5] with the Hind III / Spe I linker from pCR-Blunt II-TOPO (Invitrogen) to make pAR175. The *ATML1* promoter was excised from pAS99 and cloned into the Hind III site of pAR175 to make pAR176. The H2B-mGFP fusion protein in pAR179 was recombined into pAR176 using a gateway LR reaction to generate pAR180 *pATML1::H2B-mGFP* with Basta resistance in plants. pAR180 and pAR181 were electroporated into *Agrobacterium* and transformed into Ler, *lgo-1*, and *pATML1::KRP1* plants by floral dipping.

To create epidermal specific plasma membrane marker *pATML1::mCitrine-RCI2A* (pAR169), the mCitrine-RCI2A fusion in *pENTR-mC-RCI2A-g* [6] was recombined using a gateway LR reaction into the *pATML1* gateway expression vector pAR176. pAR169 was electroporated into *Agrobacterium* and transformed into wild type Ler and *pATM1::H2B-mYFP* plants through floral dipping. Transgenic plants were selected for Basta resistance.

LGO was overexpressed from two promoters. First, *LGO* was constitutively overexpressed from the 35S promoter. The *LGO* CDS was amplified from genomic DNA (because there are no introns) using oAR356 (5'-caccggatccATGGATCTTGAATTACTACAAGAT-3') and oAR313 (5'-agagctcTCATCTTCGAGAACAATAAGGGTA-3') and cloned into Invitrogen's pENTR D TOPO to create pAR173 (*LGO* entry clone). *LGO* from pAR173 was recombined into the 35S vector pB7WG2 [5] to create pAR174 (*35S::LGO*). Agrobacterium transformed with pAR174 were used to create transgenic Ler plants by floral dipping. Transgenic plants were selected for Basta resistance. The phenotype was strong in T1 plants, but was silenced in subsequent

generations. *LGO* was also ectopically expressed throughout the epidermis under control of the *ATML1* promoter by recombining *LGO* from pAR173 into the *pATML1* expression vector pAR176 (see above) to created pAR178 (*pATML1::LGO*). Transgenic Ler plants were created through floral dipping in Agrobacterium transformed with pAR178. Transgenic plants were selected for Basta resistance.

Scanning electron microscopy

Stage 14 flowers were prepared as described [7] and viewed on a LEO 1550 VP FESEM. Giant cells identified by their morphology and slight protrusion from the plane of the sepal were false colored in red with Photoshop CS.

Details of live imaging procedure

The conception of the live imaging experiment was based on [8,9], but the experimental details of plant growth and manipulation were altered to observe the lateral side of sepals instead of apex of the meristem. Plants were grown in soil pots covered with window screen to keep the dirt in place. Plants were imaged after bolting at least 5 cm. On the first day, at least 4 hours before imaging, the overlying flowers were removed from the inflorescence to expose the flower of interest (generally in stage 2 or 3). The older flowers on the opposite side of the meristem were removed so that the stem would lie flat against a slide. Flowers up to stage 12 were retained on the lateral sides of the inflorescence, which was important for the viability of the plant. The stem of the plant was taped to a slide such that the flower was exposed for the microscope. While the plant was in the growth room, the slide was taped to a small stake to keep the plant growing upright.

The plants were imaged on a Zeiss 510 Meta confocal laser-scanning microscope every 6 (or 12) hours. About half an hour before imaging, the plant was tipped on its side, the inflorescence was immersed in 0.02% silwet 50 μg/ml Propidium Iodide (PI, Sigma P4170-10MG), and mounted with a cover slip. The plant was placed sideways on the microscope such that the slide attached to the inflorescence was in the stage slide holder. The plants were imaged using either a 40X W-Achroplan water-dipping 0.8 NA objective, a 20X Plan Neofluar 0.5 NA objective, or a 10X Plan Neofluar 0.3 NA objective as dictated by the size of the sepal. The 40X objective was mounted in a drop of water above the cover slip. The laser power was set to 50% and the transmission to less than 10%. The transmission was set higher for non-time lapse images. YFP and mCitrine were excited at 514 nm wavelength of an argon ion laser using a HFT 458 / 514 primary dichroic and detected using a NFT 545 secondary dichroic to split the beam and 530-550 band pass filter. PI was simultaneously excited at the 514 nm wavelength and detected using the beam passing through the NFT 545 secondary dichroic and a 585-615 band pass filter. GFP was excited using a 488 nm wavelength from the argon laser and a 488 primary dichroic and detected with a 545 secondary dichroic and a 505-550 band pass filter. A 512 X 512 pixel frame was scanned at 1 μm intervals in the Z dimension. Sufficient Z sections were taken to visualize the whole sepal, which changed as the sepal grew. Additionally the zoom and objectives were adjusted during the experiment as the sepal grew such that the whole sepal could be observed. Any sepals that stopped growing during the live imaging sequence were excluded from analysis. After imaging was completed the inflorescences were dried with a kimwipe and returned to a vertical position in the growth room. All plants were grown in continuous light.

Projections of the images were made using the Zeiss LSM software (Figure S4A-B). The confocal image stacks were processed with the Amira 4.1 software (Visage Imaging, Mercury

Computer Systems). The confocal stacks were visualized using the 3D volume rendering function Voltex. Additional flowers were cropped from the image using the volume edit function, which was necessary for registration. The sepals were registered using a combination of hand alignment and the rigid Affine Registration function in Amira (Figure S4C-D). A specific angle and magnification was chosen such that the whole time-lapse sequence could be best observed. Then a single snapshot image was taken of the volume rendered sepal at each time point, generating a complete time sequence of the sepal viewed at the same angle and magnification. Cell lineages were tracked manually using colored dots in Adobe Photoshop CS as in [9,10] (Figure S4E-H). At the first time point, each cell (defined by its nucleus) was assigned an individually colored dot in a new Photoshop layer. At each subsequent time point, the set of dots was from the previous image was transferred to the new image. Correspondence between nuclei was determined by examining consecutive frames of a movie. To take into account the growth of the sample, the dots for the cells that had not divided were adjusted such that they overlie the new nucleus. After a division, two nuclei were present in the place of the original cells. The colored lineage dot was duplicated and each daughter received the same colored dot. In addition the daughter nuclei immediately following a division were outlined in white to highlight the division.

Image processing:

Segmentation of Sepals

To measure the areas of mature sepals, images were taken of mature (stage 14) sepals stained overnight in toluidine blue and photographed individually with a Zeiss Stemi SV 11 Apo

dissecting microscope and a Zeiss AxioCam HRC digital camera.

We have segmented whole sepals using ACTIWE, our C++ implementation of the Active Contours Without Edges level set based model [11]. Sepals were strongly stained producing almost completely homogeneous colors and a high contrast between background and foreground colors. This helped ACTIWE escape local minima and converge to a robust solution in a few iterations. Careful parameter tuning was necessary in a few cases of non-stained sepals to prevent over segmenting. ACTIWE automatically segments by solving the Euler-Lagrange partial differential equation corresponding to the energy model presented in [11]. We solve this geometric evolution PDE numerically using a stabilized method, allowing for easy control of the contour evolution.

The resulting average sepal areas were compared with a standard z-statistic for difference between means. For *lgo-1* versus wild type, $z=2.56$ and $p<0.05$. For wild type versus *pATML1::KRP1*, $z=7.35$ and $p<0.001$.

Segmentation of Sepal Cells

To measure cell areas, we used the epidermal specific plasma membrane marker pAR169 *pATML1::mCitrine-RCI2A*. Whole mature stage 12 sepals expressing the marker were imaged with the 20X objective and settings described above in the live imaging section. Multiple 20X 3D image stacks were taken to cover the whole sepal and projections of the stacks (Zeiss LSM software) were merged in Adobe Photoshop to produce a complete mosaic image of the sepal.

Cells in sepals were segmented using a semi-automatic image processing pipeline. We started by

denoising the gray level confocal images of the fluorescently labeled plasma membranes using a fast nonlocal means based filter [12]. Noise was significantly reduced throughout the entire images after a few passes (typically five) of the filter. The good quality of cell walls after denoising suggested the adoption of an edge detection scheme to automatically trace cell boundaries. We thus computed weighted first derivatives of the denoised images and, after thresholding them, we applied a set of mathematical morphology operators (hole filling, thinning, cleaning, and spurs removal) [13] to create one pixel wide contours delineating all cells in the sepals.

Limitations in image quality and in our algorithms required supervision to help solve erroneous segmentations. Manual editing was consistently done in binary images representing thick walls produced after morphological operations and not on the original confocal images. With minimum editing we were able to generate high quality segmentations not obtained with other tools and in a fraction of the time required by standard approaches. We validated the segmentation by visually inspecting the superposition of the segmented boundaries on the walls in the original images. Once segmented, cell sizes (e.g. area, perimeter, diameter, etc.) were computed. Except for the filtering, all processing steps were done using Matlab.

Counting Nuclei

To count cells in the sepal epidermis we imaged the equivalent epidermal specific nuclear markers pAR89 *pATML1::H2B-mYFP* for the abaxial side and pAR180 *pATML1::H2B-mGFP* for the adaxial side. Two independent programs were used to count the nuclei in sepals to provide independent validation. Method 2 by Tigran Bacarian is used in [14]. Method 1 is

described below. Both programs produced false negatives by missing nuclei and false positives by counting background as additional nuclei. Both programs produced similar results.

For method 1, we have used mathematical morphology-based approach for segmentation of cell nuclei. The algorithm work-flow consist the following procedures:

a) image pre-processing: where the 2D input image I (see Figure I1a) is filtered by morphological closing and opening with disk structuring element of size 3 pixels [15],
b) image segmentation: where the filtered image I_f is thresholded at automatically selected grey level [16],
c) image post-processing: where the segmented image I_t is filtered by morphological closing by reconstruction with disk structuring element of size 3 pixels [15].

The resulting segmentation is presented in Figure I1.

(A) 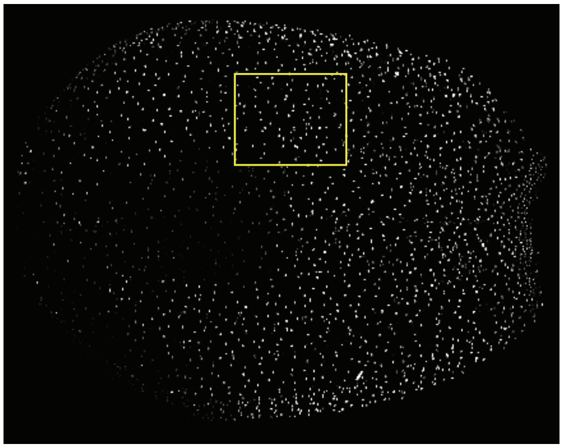

(B) (C)

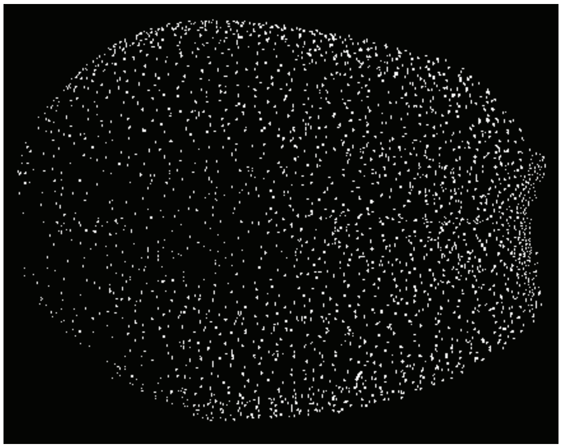 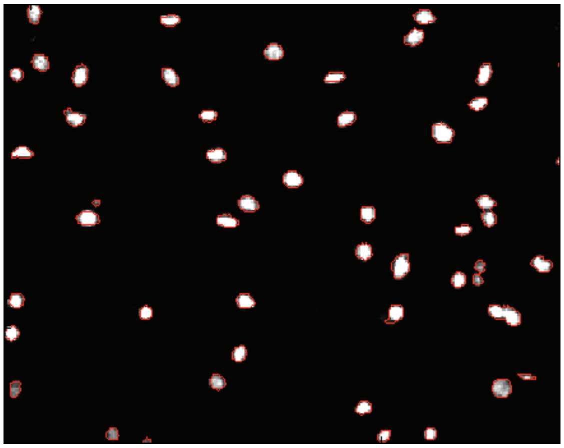

Figure I1: Nuclear counting method 1

(A) 2D confocal projection image of *Arabidopsis thaliana* adaxial sepal epidermal nuclei (*pATML1::H2B-mGFP*).

(B) Nuclei segmentation result.

(C) Zoomed region of interest from (A) with segmented nuclei boundaries outlined in red.

Modeling Supplement

Introduction

Based upon the experimental results, we have constructed a geometric based, multi-cellular model of growing and proliferating cells called the Intercalary Growth Model (IGM). The IGM represents the growing sepal starting from a meristem-like layer of generative cells. These cells divide and the daughter cells enter a program of endoreduplication, wherein they decide whether to divide or endoreduplicate, with a probability dependent on the number of cell cycles the cell has completed. The decision to endoreduplicate or divide is based upon probabilities, which are determined by a simple population model, the description of which follows.

Population model

Consider the conceptual model in Figure M1. A 2C cell makes the random decision with probability p_1 to endoreduplicate to 4C or with probability $1-p_1$ to divide and remain 2C. Once a cell has decided to endoreduplicate, it continues to do so and cannot resume mitotic divisions [17]. In the next cell cycle, all of the 4C cells endoreduplicate to 8C. Simultaneously, the 2C cells commit to endoreduplicate to 4C with probability p_2 and the remaining cells divide. In the final patterning cell cycle, those cells that decided to endoreduplicate earliest become the 16C giant cells, while those cells that continue to divide become small cells. The specialized division patterns of stomatal development follow at the end of this process and 2C cells continue to divide with probability p_s.

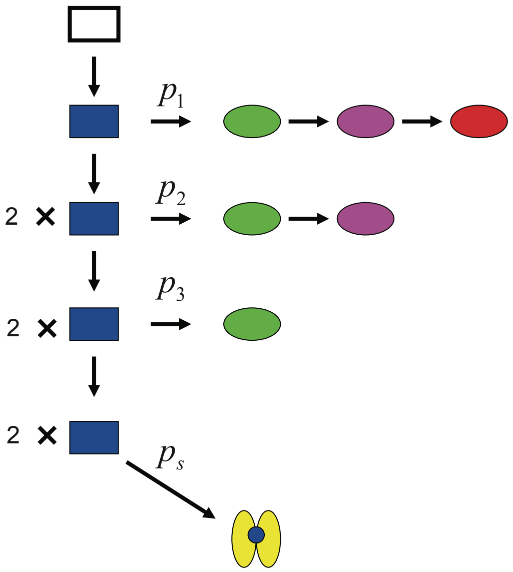

Figure M1: Population dynamics, showing the endoreduplication program.

There are 4 cell cycles, within which cells can endoreduplicate for a maximum of 3 times. These occur with probabilities p_1, p_2, p_3. At the final stage a fraction of cells go into the stomatal lineage and divide. The remaining 2C cells do not divide. The color indicates the ploidy of the cell: red = 16C, magenta = 8C, green = 4C, blue = 2C, and yellow = stomatal lineage

Hence if we begin with a template of N_0 cells, at the end of 3 cell cycles, we are left with the following proportions of different cell types.

$$N_{16C} = p_1 N_0, \quad (1)$$

$$N_{8C} = 2p_2(1-p_1)N_0,$$

$$N_{4C} = 4p_3(1-p_2)(1-p_1)N_0,$$

$$N_{2C} = 8(1+p_s)(1-p_3)(1-p_2)(1-p_1)N_0,$$

where p_1, p_2, p_3 are the probabilities to endoreduplicate for the first three cell cycles, and p_s is the probability for a stomatal division, which occurs at the last stage. The total number of cells is given by,

$$N_T = fN_0, \qquad (2)$$

where $f = p_1 + 2p_2(1-p_1) + 4p_3(1-p_2)(1-p_1) + 8(1+p_s)(1-p_3)(1-p_2)(1-p_1)$.

We determined that $N_T = 1600$ by counting the cells in the abaxial sepal epidermis using nuclear segmentation (Figure S2J). We also have found that 29% of the total cells are stomatal by manually counting. Hence $16(p_s)(1-p_3)(1-p_2)(1-p_1)N_0=464$. In plant organs such as the sepal, both sides of the organ are covered with epidermal cells (as defined by both morphology and gene expression patterns). Therefore, the measured ploidy distribution of cells in the mature sepal epidermis (Figure 1D) includes cells from both sides of the sepal; however, the cell size pattern only occurs in the abaxial side. The cells in the adaxial (back) epidermis of the sepal are all 2 or 4 C. Therefore, in the measurement for the ploidy levels, the total number of 2C, 4C cells acquire a contribution from the 2C and 4C cells from the back of the sepal, where the total number of epidermal cells on the back is N_{Tb}=2800 (Figure S2J). The measured mean ploidy distribution is 16C=1%, 8C=6%, 4C=32%, 2C=61% (Figures 1D and 4I). We explored the parameter space because the ratio of 2C to 4C cells on the back of the sepal is unknown. The first

question that arises is whether the cells from the back of the sepal can all be only 2C cells. The following argument suggests that this cannot be the case. Let all of the cells from the back be 2C cells, then, the total 2C cells N_{2C}=8(1+p_s)(1−p_3)(1−p_2)(1−p_1)N_0+2800. Now dividing the ratios of 16C to 8C and 8C to 4C, we obtain the following two equations for the probabilities p_3,p_2 in terms of p_1,

$$p_2 = \frac{3p_1}{1-p_1}, \qquad p_3 = \frac{2.67p_2}{1-p_2}, \tag{3}$$

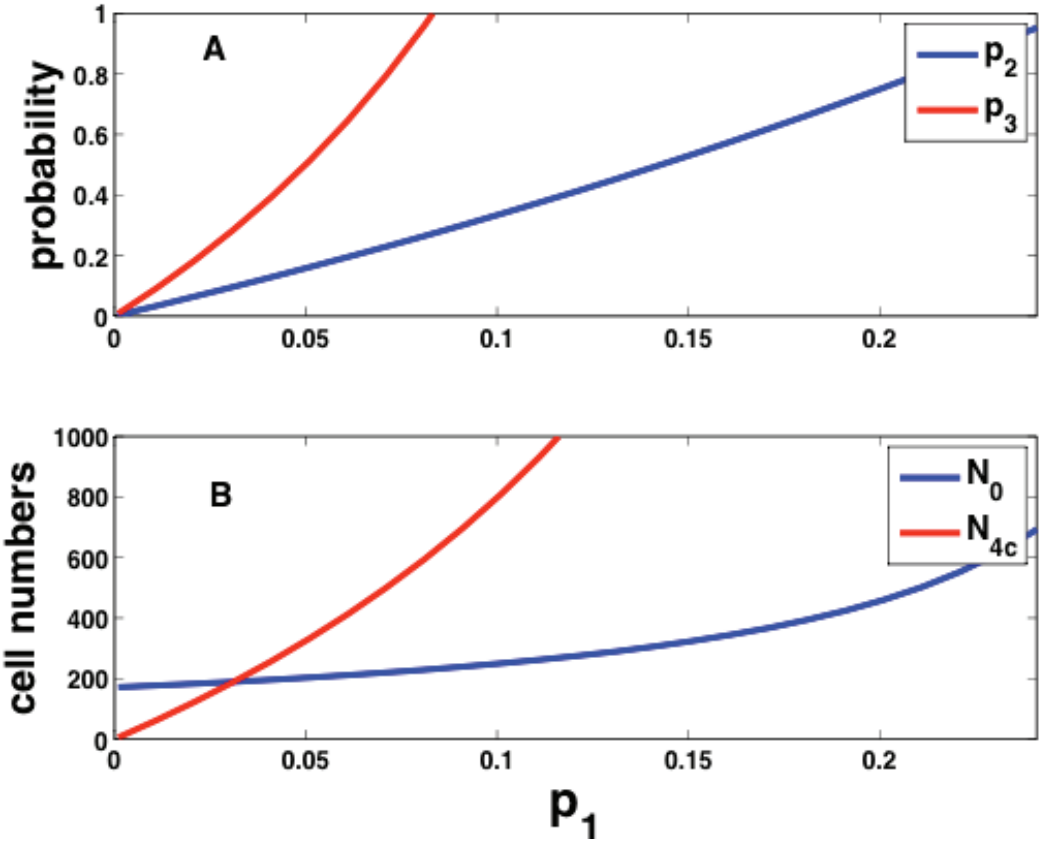

Figure M2: Probabilities p_2,p_3, cell numbers N_0,N_{4c} as a function of p_1 when N_{4cb}=0.

In Figure M2A, we plot p_2,p_3 as a function of p_1 when all cells on the back of the sepal are 2C (N_{4cb} = 0), which clearly shows that p_1 must be restricted to less than 0.07 for realistic values of p_3. In the lower panel, we plot N_0 obtained from Equation 2, and N_{4c}, from Equation 1. From the observed ploidy distribution, the total number of 4C cells should be approximately 31% of N_{Tf}+N_{Tb}=4400 cells, which corresponds to ~1364 cells. However, from the plot we see that this cell number is never reached, indicating that our hypothesis that all the cells from the back are 2C is incorrect. Hence we assume that N_{2cb}+N_{4cb}=2800, where the 2C and 4c cells from the back are represented by N_{2cb},N_{4cb}.

We have optimized the parameters, p_1, p_2, p_3, N_{2cb}, by fitting the ploidy distribution. We first use a standard implementation of a genetic algorithm (GA) [18] to provide a best beginning guess for the parameters, and then use a local optimization algorithm to obtain the best fit. We start the GA with a random population of individuals (100), and perform the following steps: Tournament Selection: 10 members are randomly selected from the population of 100, and ranked according to their fitness. The best member always gets selected, and the remaining members are selected based upon roulette selection. At the end of the selection step, 2 parents are retained. Crossover: The selected parents are crossed over [19], using an arithmetic mean λ=0.7, to give two children. Then a selection of two members is made from the parents and children, by first choosing the best amongst all of them and then using roulette selection among the remaining 3. Mutation of one parameter is randomly selected and mutated. For the local optimization method, we used the built in MATLAB implementation of non-linear least squares optimization routine (lsqnonlin).

We find by running the search for 1000 times a parameter set with means,

$p_1 = 0.1361 \pm 0.0075, p_2 = .4727 \pm 0.0296, p_3 = .1892 \pm 0.1981, N_{2cb} = (0.5428 \pm 0.0564) * 2800.$

One member of this parameter set that we chose is,

$p_1 = 0.1172, p_2 = .3985, p_3 = .6702, p_s = 0.4416$, for which the corresponding cell numbers are

$N_0 = 375, N_{2cb} = 1926, N_{4cb} = 874, N_{2cf} = 758, N_{4cb} = 534, N_{8cf} = 264, N_{16cf} = 44$.

Corresponding to the measured ploidy levels, the average sepal contains 44 giant cells with 16C ploidy and 264 cells with 8C ploidy. Since the 2C and 4C cells from the back of the sepal contribute to the 2C and 4C populations, there is considerable variance in these cell numbers. The 8C and 16C numbers are tightly constrained. This can be understood as follows: The total of N_{4C} cells, from the front and back is 31% of the 4400 cells ≈ 1400. We use the value of the number of 4C cells for the front of the sepal from Equation 1 to give

$$p_3(1-p_2)(1-p_1)N_0 + 2800 - N_{2Cb} \leq 1400 \tag{4}$$

The above equation implies that $N_{2Cb} \geq 1400$. Taking the limiting case, we get, $N_{2Cb} = 1400$, for p_3=0. Now the total number of 2C cells is 61% which corresponds to 2684, hence from Equation 1, using the expression for the number of 2C cells in the front of the sepal, and accounting for the stomatal cells, from $16(p_s)(1-p_3)(1-p_2)(1-p_1)N_0 = 464$, we obtain,

$$1052 = 8(1-p_2)(1-p_1)N_0, \tag{5}$$

For the template cells, we have from Equation 2,

$$N_0 = \frac{1368}{8(1-p_2)(1-p_1)+2p_2(1-p_1)+p_1} \tag{6}$$

From the 1%, 6% fraction of 16C, 8C cells, respectively we obtain a constraint between p_1,p_2 which is, $\frac{N_{8c}}{N_{16c}} = \frac{2p_2(1-p_1)}{p_1} \approx 6$, which implies that $p_2 = \frac{3p_1}{1-p_1}$. Finally from Eq. 5, 6, we obtain an expression for p_1, $p_1 \leq 0.1447$, which therefore limits, p_2<=0.5. However, since the lower limit for p_3=0, this implies that all the 4C cells are contributed from the back of the sepal. Increasing p_3 gives more 4C cells, which then changes the values of the other probabilities, to maintain the correct ploidy distribution.

Using the parameter set, $p_1 = 0.1172, p_2 = .3985, p_3 = .6702, p_s = 0.4416$ we ran a stochastic simulation (Monte Carlo) of the population model, starting with a template of N_0=375 cells, and obtained the ploidy levels displayed in Figure M3. As we would expect the ploidy levels agree well with the experimentally observed ones, and hence the chosen parameter values are reasonable.

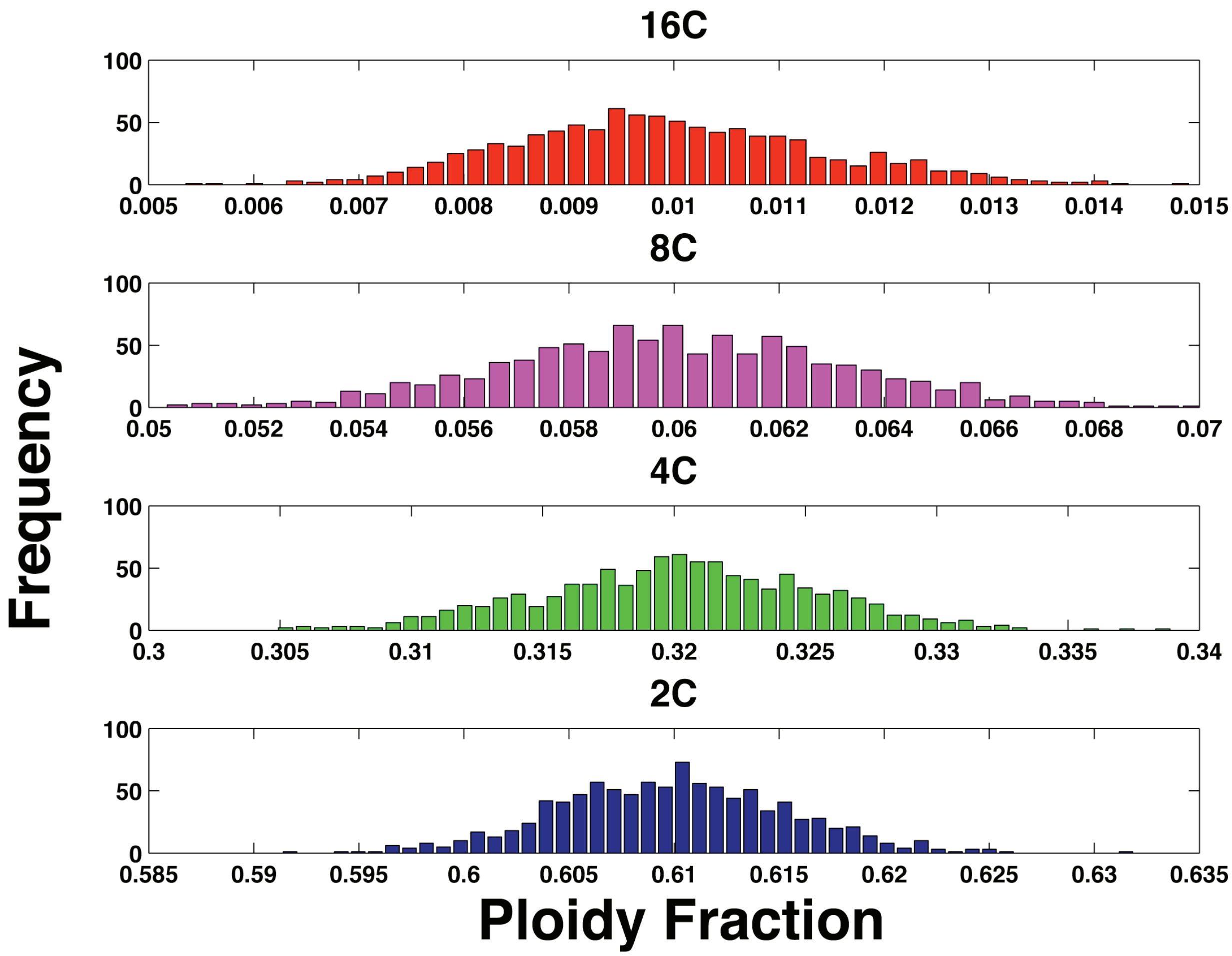

Figure M3: Monte-Carlo simulation of the population model showing the measured average ploidy levels.

Intercalary growth model (IGM)

The IGM model follows from the assumptions given below.

(1) First we assumed that all cells grow such that distortions in the sheet of cells [20] are prevented. Cell growth is modeled such that cells do not slip with respect to each other. This can be simply implemented if it is assured that the sum of the growth rates of two recently divided daughter cells, which previously shared a common cell wall with its neighbor, is equal to the

growth rate of the neighbor. Cells grow anisotropically such that the vertical growth rate is greater than the horizontal growth rate.

(2) Second, we include variability in the duration of the cell cycle as observed in the live imaging data (see next section).

(3) Third, we assumed that the decision to endoreduplicate was random with a probability that depended on the cell cycle. The probabilities used were determined from the ploidy data using the population model (see previous section).

(4) Fourth, we assumed that once a cell enters endoreduplication it can no longer divide and continues to endocycle [17].

(5) Fifth, we assumed that the total number of endocycles undergone by the most highly endoreduplicated giant cell represents the maximum number of patterning cycles that the tissue undergoes regardless of whether those cycles are mitotic cycles or endocycles. Thus, we limited the number of cell cycles to 3 corresponding with the three endocycles required to reach 16C. At the end of these divisions, remaining 2C cells enter the stomatal pathway with probability p_s (Figure M1). These cells would continue to undergo a minimum of one more division, which is not modeled.

(6) Sixth, without noise in the cell cycle time, we assume that a cell would double its area in each cell cycle whether mitotic or endocycle. Upon the addition of noise as sampled from distributions derived from live imaging, cells no longer grow to exactly twice their area.

(7) Seventh, we assumed that cell division is not precisely symmetric, with daughter cells randomly partitioned with some noise as determined from imaging data (see next section).

(8) Eight, for the cells that divide, only horizontal or vertical division planes are allowed. The plane is chosen that produces daughter cells with the length to width ratio closest to 2:1. This

prevents cells form becoming abnormally long or wide. Both horizontal and vertical divisions are observed in the imaging data (Movies S1-S3).

The intercalary growth (IG) model seeks to describe the cell size patterning of the entire sepal: from initiation on the side of the floral meristem to the termination of growth in the mature sepal. The live imaging suggests (Figure 3A-F) that a group of cells from the bottom of the sepal give rise to the rest of the sepal cells through repeated divisions. We simplify this concept to a "generative layer" of cells that continuously divide and give rise to 2C daughters. This generative layer is similar in concept to the proposed intercalary meristem of grass leaves. Each of the 2C daughter cells is ready to enter the 3 cell cycle/endoreduplication program.

The two key factors that account for the variability of the sizes of cells with the same ploidy. (i) First, in live imaging, we observe that cells do not divide into two equal halves (Figure M4). We find that the standard deviation in daughter cell areas of non-stomatal division is about 10%. Therefore, we divide cells into two halves ± 10%.

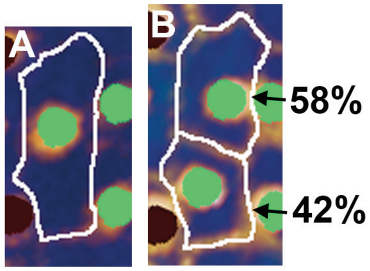

Figure M4: Daughter cell sizes are unequal, with noise of 10%

The cell outlined in white (A) divides within the following 6 hours to produce the daughter cells outlined in (B). Note that the new cell wall does not partition the daughter cell areas equally. The upper daughter is 58% of the total cell area and the bottom cell is 42%.

(ii) Second, from the live imaging we observe that cell cycles are asynchronous (Supplemental Movies S1-S3). We quantified the cell cycle times of individual cells in the wild type live images (as the intervals between one division and the next, or the start of imaging and the first division) (Figure 3I). We have fitted this cell cycle time distribution with a Weibull distribution, described by

$$f(x|a,b)=ba^{-b}x^{b-1}exp-(\frac{x}{a})^{b}.$$

The fitted distribution is shown in Figure 3I, with the shape parameter b=2.1, and the scale parameter a=4.75, and with the 95% confidence levels (δa 4.47,5.04, δb 1.92, 2.3). The distributions for *lgo-1* and *pATML::KRP1* were measured from live imaging data and fit to the Weibull distribution, (Figure 6C-D; a=4.24 and b=2.03 for *lgo-1*; a=5.096 and b=2.45 for *pATML::KRP1*). As can be seen, the cell cycle times for *lgo-1* and *pATML::KRP1* are slightly shorter and longer respectively compared to the wild type cell cycle times (Twildtype=4.2, T*lgo*=3.76, T*patml::krp1*=4.52 (in units of 6 hrs)). To test whether the cell cycle distributions were significantly different, we used the two sample Kolmogorov-Smirnov test (K-S) at the 5% significance level. K-S (wild type, *pATML::KRP1*) rejected the null hypothesis of similar distributions with a p-value=0.049; the K-S (wild type, *lgo*) did not reject the null hypothesis with a p-value=0.1624. Therefore, to further test how significantly different the mean values are, for this case, we used the two tailed t-test, which rejected the null hypothesis of equal mean values with a p-value=0.0067.

We now use the flowchart (Figure M5) to describe the model.

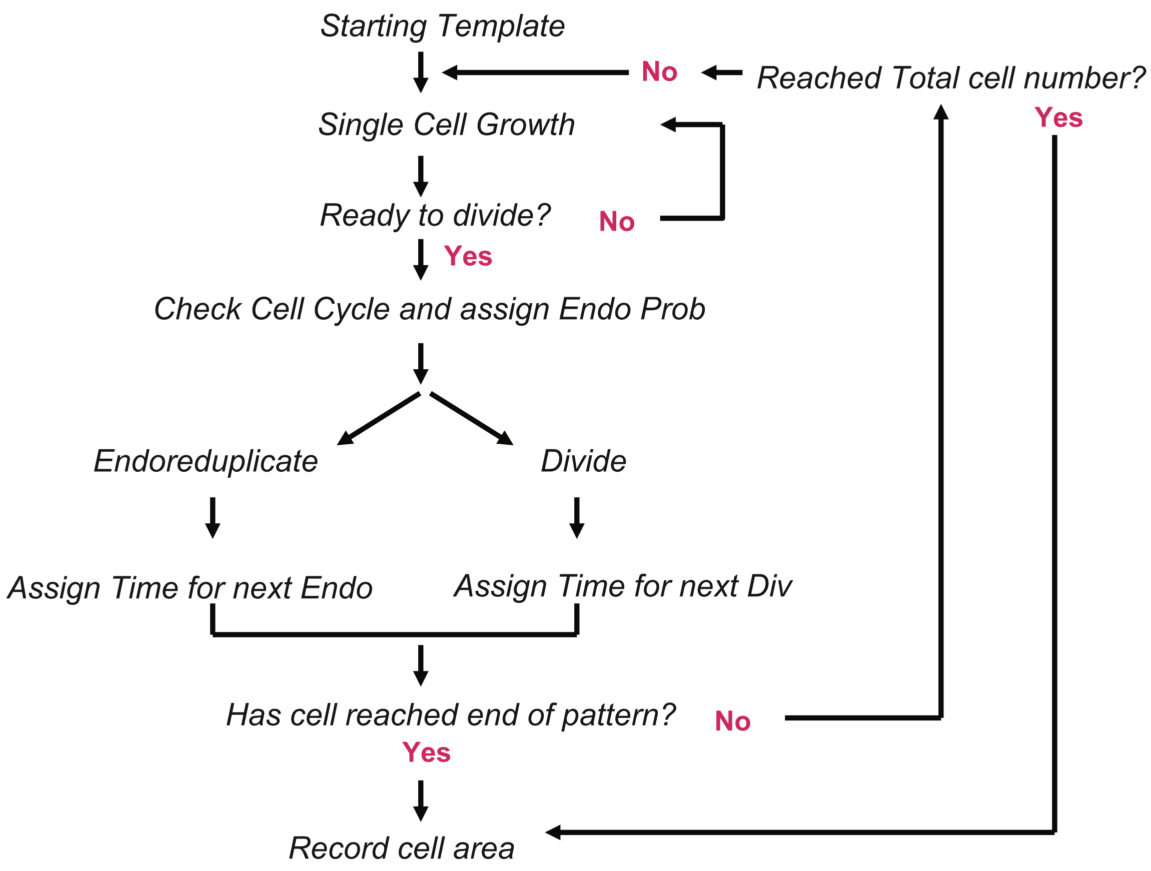

Figure M5: Flow chart for the Intercalary Growth model.

Starting Template

To determine the starting template for the model, we imaged the emergence of the sepal from the flank of the floral meristem. We found that the sepal emerges from a set of cells about 8 cells wide (Figure 3A-B). This is consistent with sectoring data that showed the sepal emerges from a line of 8 cells in the floral meristem [21]. Therefore, the starting template is assumed to be a file of 8 cells, which we call the generative layer (Figure 3G).

We assume that this layer of cells proliferates and gives rise to cells, each of which will then go into the endoreduplication program:

- If the division of a generative cell is vertical, the upper daughter is competent to enter the endoreduplication patterning program, while the lower daughter maintains generative identity.
- If the division of a generative cell is horizontal, both cells remain in the generative layer and continue to proliferate.
- The cell, which is ready to enter the endoreduplication program, now undergoes repeated divisions or endocycles as discussed earlier.

Single Cell Growth

Each cell's length and width are made to grow exponentially in time (with the exponential growth rate parameters, $\lambda_{vertical}$ =1 ; $\lambda_{horizontal}$ = 0.35], at rates that are parameterized to give around ~125 generative layer cells at the end of the simulation time, which is chosen to give about ~1400 cells (reduced from ~1600 to account for the additional stomatal cells which will finally populate the sepal, not shown in the model). The neighboring cells grow at the same rate, to prevent cells from slipping past each other. Cells with longer cell cycles reach larger sizes before dividing than cells with shorter cell cycles (Figure 2C-D and S1). Therefore, cell cycle times are directly related to the area the cell reaches by the end of the cell cycle. In this model we assume that on average the cell divides when it reaches roughly twice its starting area, which is also when the next cell cycle begins. Since cell divisions are observed to occur asynchronously (Figure 2C and Movies S1-3), this naturally translates into cells choosing different areas to which to grow, which are sampled from the observed distribution in cell cycle time. We

therefore sample from a Weibull distribution which is centered at *area*=2 (we have chosen our template 2C cells to have a mean area=2). At the beginning of each cell cycle, the cell selects a cell cycle time and corresponding area from this distribution $f(T)$. This area is added to the initial area of the cell at the start of the cell cycle to determine the final area of the cell at the end of the cell cycle immediately before division or the start of the next endocycle. Hence, at the end of the cell cycle, a reasonable assumption is that the distribution of 4C cell areas is from the sum of two random vectors sampled from $f(T)$. The sum can in turn be sampled from the convolution of $f(T)$, $g(T) = f(T) \otimes f(T) = \int_0^T f(T-t) f(t) dt$. We now make a simple assumption, namely that endocycles behave in a similar way to cell cycles, *i.e.*, endocycle times are sampled from the continuation of the cell cycle. Hence the 8C cell cycle time would be sample from the distribution, $h(T) = g(T) \otimes g(T)$. A similar approach is taken for the *lgo-1* and *pATML::KRP1* cell cycle distributions. In Figure M6 we plot the distributions from which we sample for the cell cycle and endocycle times.

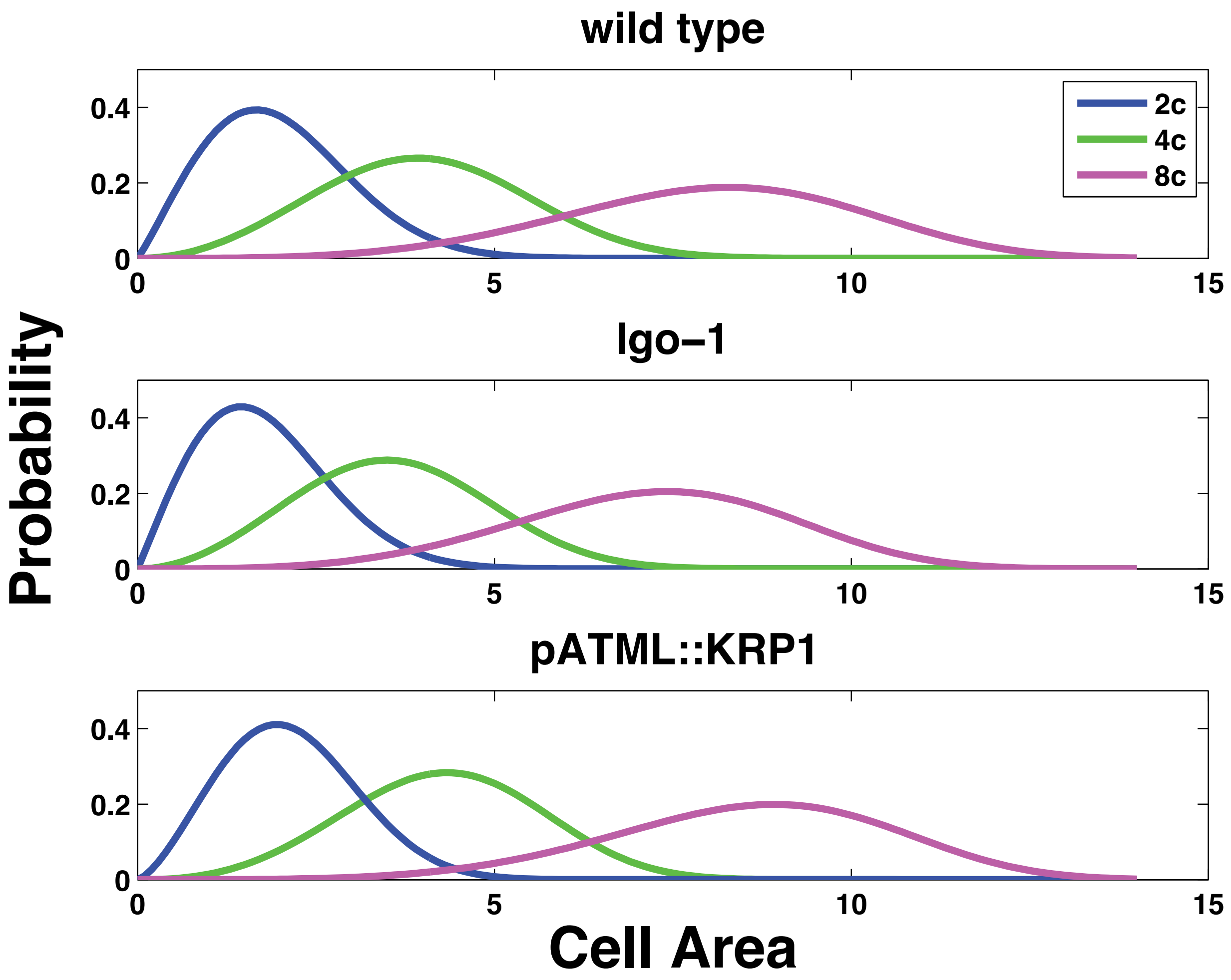

Figure M6: The sampling distributions of cell cycle and endocycle times for 2C, 4C and 8C cells.

Decision to divide or endoreduplicate

When cells complete their cell cycle and reach their target area, the cells decide to divide or endoreduplicate. The probability is chosen based on how many cell cycles the cell has undergone. For example, if the cell is a direct descendant of the generative cell, p_1 is used. If the cell decides to endoreduplicate, then the first endocycle corresponds to an area, which is chosen from the distribution above.

Record Growth when cells terminate the cell cycle

Each descendent of the generative layer has only three cell cycles, either mitotic or endocycles, before it terminates cycling. It is at this point that cell stops growing, while its neighbors may continue to grow till they stop cycling as well. In the visualization of our model, to prevent cells from overlapping one another, which would occur if some cells stop growing, we allow all the cells to continue to grow; although the final area was recorded at the end of the cell cycle. The output of the program is shown in Figure 3G. At each iteration of the simulation cycle, we record cell areas for cells that have reached the end of their program. We stop sepal growth when we reach approximately 1400 cells, and record all cell areas. These areas are then used to compute a histogram of cell areas as shown in Figure 3K, with the contribution from different ploidy of cells shown in different colors.

Validation of Model to *in vivo* data

In this section we analyze the distribution of cell areas obtained from confocal imaging data, (Figure 3J and 6A) and compare it to the area distributions obtained through simulation (Figure 3K and 6B). The data from the simulation was obtained by Monte Carlo simulations (we run the model 10 times), and recording the areas. The data in both cases has first been normalized to unit mean, and binned in a logarithmic scale (base 2).

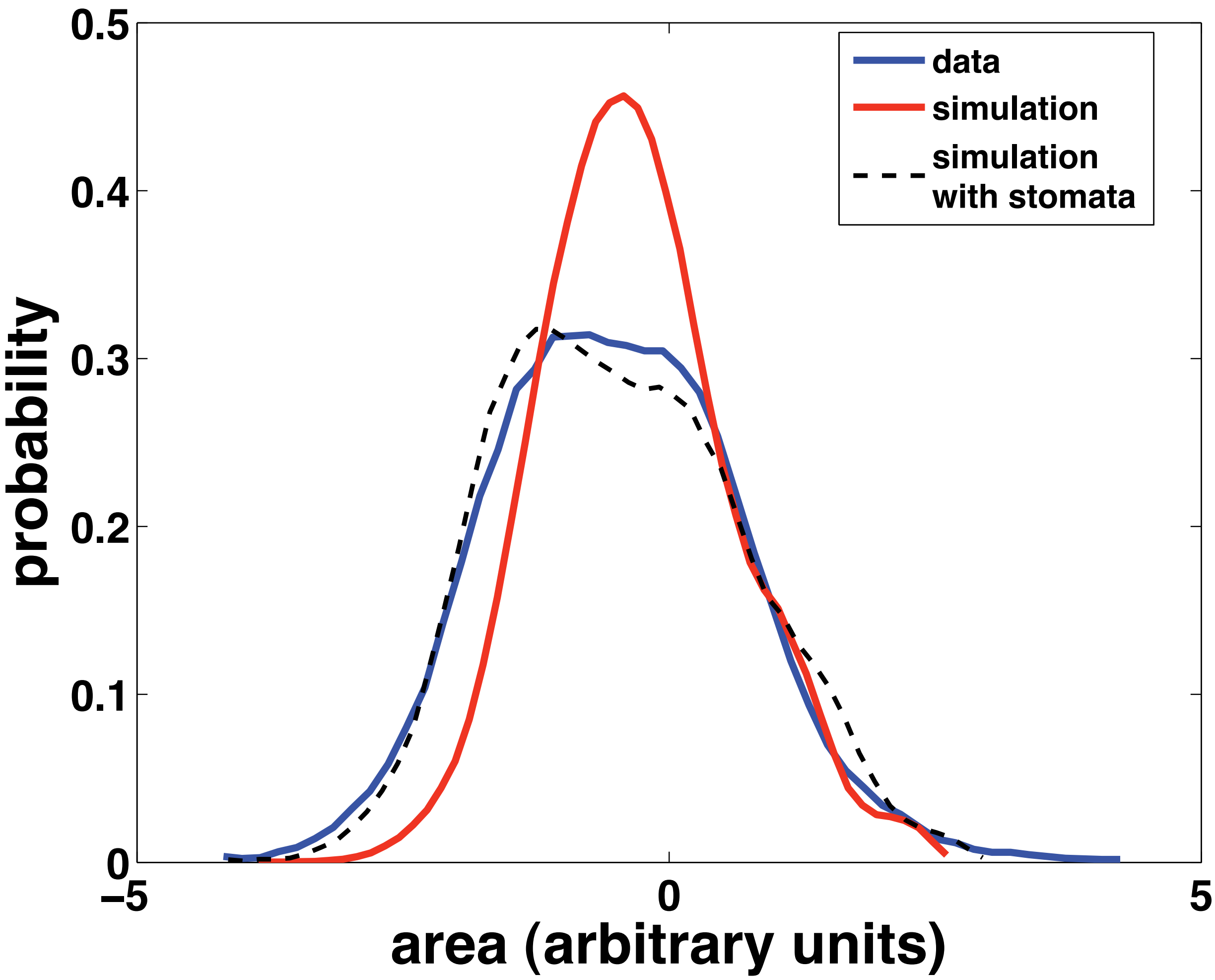

Figure M7: Comparison of wild type *in vivo* data with *in silico* simulations

Histogram of cell areas for the experimental data, as well as from the simulation.

When we include extra stomatal divisions., the *in silico* cell size distribution with stomata is not significantly different from the wild type *in vivo* distribution.

Figure M7 (blue line) outlines the histogram of cell areas (with unit mean) obtained from the imaging data. We note that the distribution is fairly broad, as compared to the distribution obtained from the simulation (red line), which seems to be underestimated for the 2C cell areas. In Figure M8 we display the empirical cumulative distribution function, of both the experimental

as well as the simulation, which looks reasonably similar. To statistically compare whether the distributions are similar we ran the Kolmogorov-Smirnov (K-S) test, which gives a p-value 1.94e−45 and $KSstat$=0.1389, which rejects the hypothesis that the two distributions are similar.

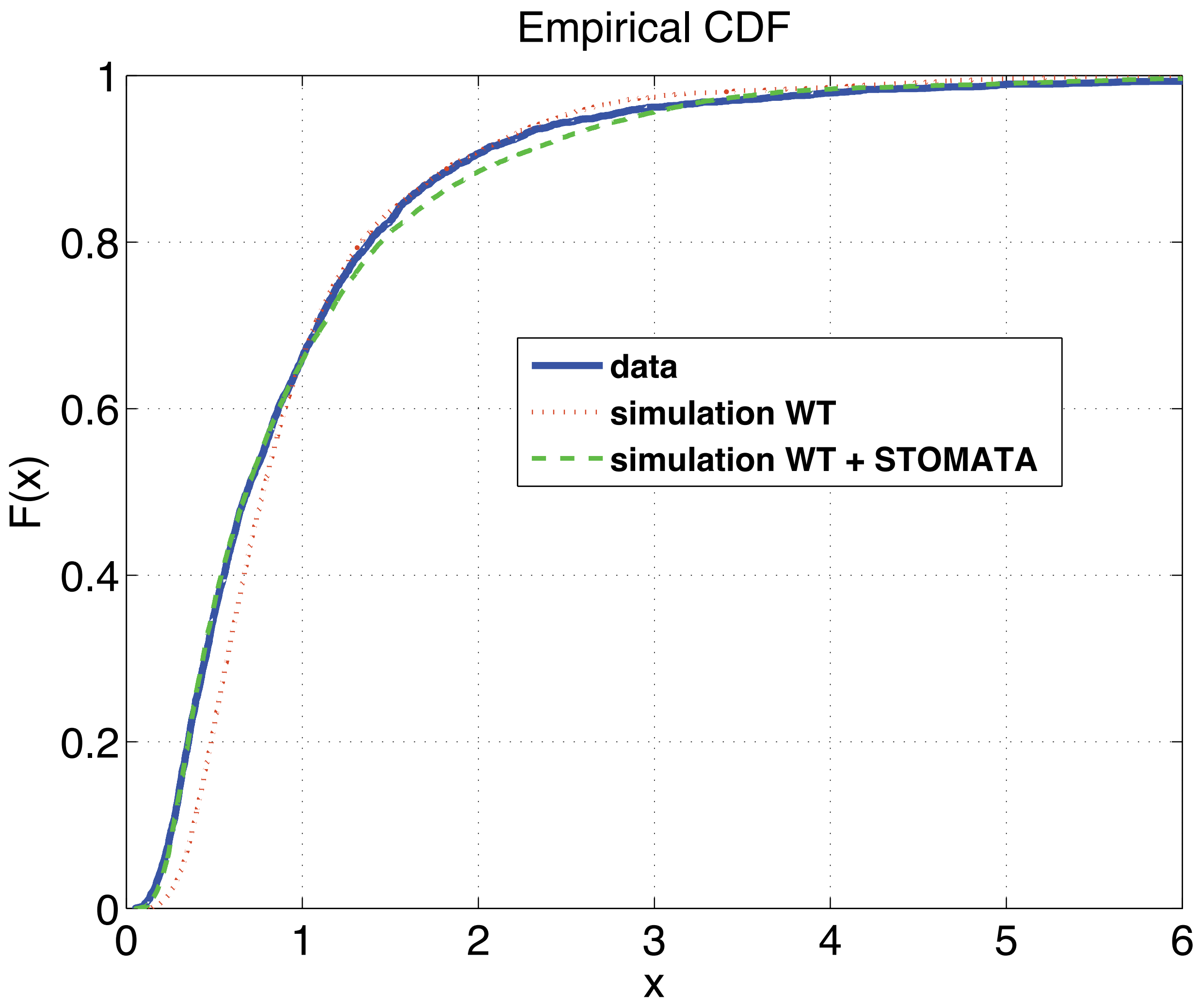

Figure M8: The computed cumulative distribution function for the distributions of the data, simulation (WT) and the simulation (WT+stomatal divisions).

This begs the question as to why is the experimental data is distributed more broadly than data obtained from the simulation? To understand the discrepancy of why the experimental distribution is higher at lower values of area, we ran the model and assumed stomatal divisions occur, as the last stage of sepal development. After every simulation of the sepal, we select 2C cells with probability p_s =0.4416, and divide them into two stomatal lineage cells to generate approximately 29% stomatal cells as observed. Thus the area distribution of 2C cells gets spread out towards lower values.

In Figure M7, the distribution of the simulation, which takes into account the stomatal divisions is shown (black dashed line). The distribution is broader at low values of area, as compared to a simulation where stomatal divisions are not included. We re-ran the K-S test between the data and the simulation taking into account the stomatal divisions, with the p-value 0.0774 and *KSstat*=0.0242, which does not reject the hypothesis that the two distributions are similar. Hence the chief determinant in the variation in the histogram of areas, is due to the stomatal divisions which increases the left tail. This can also be seen in the comparison of the empirical cumulative distribution functions between the data and the simulation including the stomatal divisions, which is more similar (Figure M8).

We also compared the cell size distribution of the model for *pATML1::KRP1* with the in vivo data. One difference between the model and the data is that the data has an extended tail of large cells (Figure 6A to the right on the graph). We tested whether altering the parameters of the model could produce this tail. We compared the distributions of cell areas for *pATML::KRP1*, obtained though simulation with the data for two cases:

(i) the *pATML::KRP1* parameters are as obtained from the measured cell cycle data.

(ii) the cell cycle length for 2C cells is noisier as well as slightly increased from the measured value and the length of the endocycles for16C cells is noisier as well as slightly increased.

In Figure M9, the cell area distributions for the data (blue curve) and simulation including stomatal divisions (red curve) differ significantly at both lower as well as higher areas. We now repeat the simulation taking into account stomatal divisions but with two assumptions. (1) 2C cell cycles are noisier and also slightly larger, (2) 16C endocycles are also noisier and considerably larger. The black curve is the result of including these assumptions into the model. Increasing the length of the 16C endocycle (black curve, right hand side green arrow) has the effect of increasing the tail of the distribution towards higher areas, and making it noisier, which has the obvious effect of spreading out the tail. This behavior better matches the *in vivo* data suggesting that overexpression of *KRP1* also increases endocycle length. On the other hand making 2C cell cycles noisier, spreads out the left side of the distribution to include even smaller areas. Hence the final distribution shifts towards the left and also has a small tail; these features are shared with the observed distribution.

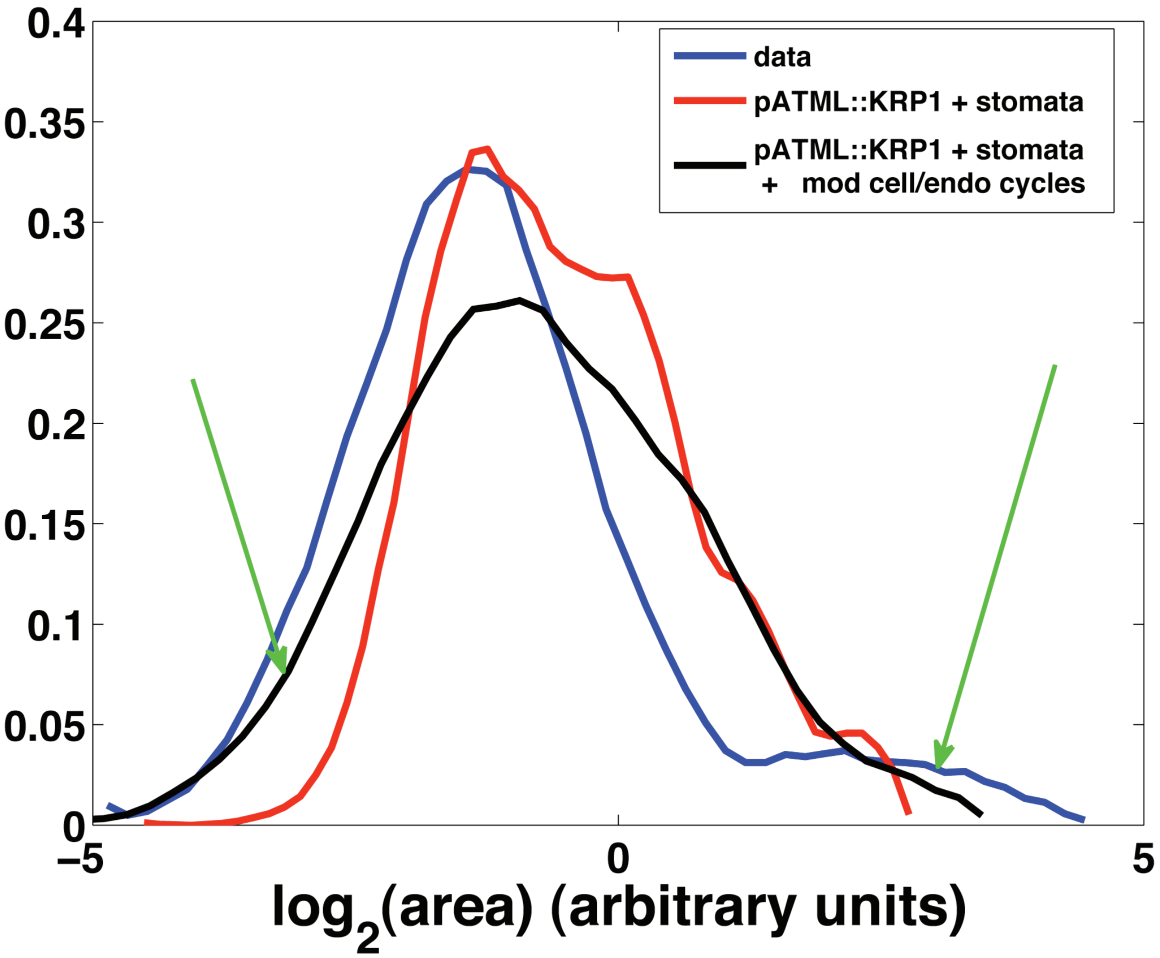

Figure M9: The distributions of the cell areas for modifying cell cycle and endocycle times for *pATML::KRP1*.

Difference between the IGM and the population model

Three important differences distinguish the IG model from the population model. First, the population model only addresses endoreduplication and does not explicitly describe cell size at all. As we show in Figure 3H-K, endoreduplication alone is insufficient to produce the cell size distribution. The cell cycle time is critical for generating the cell size pattern and this is only taken into account in the IG model. Second, the IG model addresses the spatial constraints on

growth posed by cell walls that prevent one cell from slipping relative to another. Third, the IG model describes the development of the whole sepal. If the population model is applied to an expanding template, it can only generate a region of the sepal and is insufficient to generate the whole sepal given the experimental data showing the starting number of cells in the sepal primordium.

All simulations were done in MATLAB (The MathWorks, Inc.)

Supplemental References

1. Lukowitz W, Gillmor CS, Scheible WR (2000) Positional cloning in Arabidopsis. Why it feels good to have a genome initiative working for you. Plant Physiol 123: 795-805.
2. Bemis SM, Torii KU (2007) Autonomy of cell proliferation and developmental programs during Arabidopsis aboveground organ morphogenesis. Dev Biol 304: 367-381.
3. Sessions A, Weigel D, Yanofsky MF (1999) The Arabidopsis thaliana MERISTEM LAYER 1 promoter specifies epidermal expression in meristems and young primordia. Plant J 20: 259-263.
4. Boisnard-Lorig C, Colon-Carmona A, Bauch M, Hodge S, Doerner P, et al. (2001) Dynamic analyses of the expression of the HISTONE::YFP fusion protein in Arabidopsis show that syncytial endosperm is divided in mitotic domains. Plant Cell 13: 495-509.
5. Karimi M, Inze D, Depicker A (2002) GATEWAY((TM)) vectors for Agrobacterium-mediated plant transformation. Trends Plant Sci 7: 193-195.
6. Thompson MV, Wolniak SM (2008) A plasma membrane-anchored fluorescent protein fusion illuminates sieve element plasma membranes in Arabidopsis and tobacco. Plant Physiol 146: 1599-1610.
7. Roeder AHK, Ferrandiz C, Yanofsky MF (2003) The role of the REPLUMLESS homeodomain protein in patterning the Arabidopsis fruit. Curr Biol 13: 1630-1635.
8. Heisler MG, Ohno C, Das P, Sieber P, Reddy GV, et al. (2005) Patterns of auxin transport and gene expression during primordium development revealed by live imaging of the Arabidopsis inflorescence meristem. Curr Biol 15: 1899-1911.

9. Reddy GV, Heisler MG, Ehrhardt DW, Meyerowitz EM (2004) Real-time lineage analysis reveals oriented cell divisions associated with morphogenesis at the shoot apex of Arabidopsis thaliana. Development 131: 4225-4237.

10. Reddy GV, Meyerowitz EM (2005) Stem-cell homeostasis and growth dynamics can be uncoupled in the Arabidopsis shoot apex. Science 310: 663-667.

11. Chan TF, Vese LA (2001) Active contours without edges. Ieee Transactions on Image Processing 10: 266-277.

12. Darbon J, Cunha A, Chan TF, Osher S, Jensen GJ (2008) Fast nonlocal filtering applied to electron cryomicroscopy. IEEE ISBI: 1331 - 1334.

13. Soille P (2007) Morphological Image Analysis: Principles and Applications: Springer.

14. Gor V, Elowitz, M, Bacarian, T and Mjolsness, E (2005)Tracking Cell Signals In Fluorescent Images. Proceedings of the 2005 IEEE CVPR pp. 142-142.

15. Serra J (1982) Image Analysis and Mathematical Morphology. New York: Academic Press.

16. Otsu N (1979) Threshold Selection Method from Gray-Level Histograms. Ieee Transactions on Systems Man and Cybernetics 9: 62-66.

17. Kowles RV, Yerk, GL, and Phillips, RL (1992) Maize endosperm tissue as an endoreduplication system. Genetic Engineering 14: 65-88.

18. Goldberg DE (1989) Genetic Algorithms in Search, Optimization, and Machine Learning: Addison-Wesley.

19. Herrera F, Lozano M, Verdegay JL (1998) Tackling real-coded genetic algorithms: Operators and tools for behavioural analysis. Artificial Intelligence Review 12: 265-319.

20. Traas J, Hulskamp M, Gendreau E, Hofte H (1998) Endoreduplication and development: rule without dividing? Curr Opin Plant Biol 1: 498-503.

21. Bossinger G, Smyth DR (1996) Initiation patterns of flower and floral organ development in Arabidopsis thaliana. Development 122: 1093-1102.
